# ENO1-related gene signature predicts prognosis and therapeutic response in diffuse large B-cell lymphoma

**DOI:** 10.3389/fimmu.2025.1644020

**Published:** 2025-10-23

**Authors:** Wenli Yan, Xiaoxi Liu, Beibei Gao, Shanshan Zhang, Jinhong Ren, Yang Lu, Limei Ai, Jinsong Yan, Haina Wang

**Affiliations:** ^1^ Department of Hematology, Liaoning Key Laboratory of Hematopoietic Stem Cell Transplantation and Translational Medicine, The Second Hospital of Dalian Medical University, Dalian, China; ^2^ Liaoning Medical Center for Hematopoietic Stem Cell Transplantation, the Second Hospital of Dalian Medical University, Dalian, China; ^3^ Department of Hematology, The First Affiliated Hospital of Jinzhou Medical University, Jinzhou, China; ^4^ Shanxi Key Laboratory of Innovative Drug for the Treatment of Serious Diseases Basing on the Chronic Inflammation, College of Traditional Chinese Medicine and Food Engineering, Shanxi University of Chinese Medicine, Taiyuan, China; ^5^ Department of Hematology, Chaoyang Central Hospital, Chaoyang, China

**Keywords:** DLBCL, ENO1, PABPC4, prognosis, therapeutic response

## Abstract

**Purpose:**

Alpha-enolase (ENO1), the enzyme catalyzing 2-phosphoglycerate conversion to phosphoenolpyruvate, is highly expressed in diffuse large B-cell lymphoma (DLBCL) and correlates with adverse clinical outcomes. Thus, understanding the relationship between ENO1-related gene (ERG) network and DLBCL is imperative. Here, we integrated multi-omics profiling (RIP-seq, RNA-seq, and protein interactome analysis) to identify ERGs and established a prognostic model by machine learning algorithms.

**Methods:**

We identified eleven hub genes (CHERP, SYNE2, INTS1, FAP, MMP9, LRP5, RBM8A, PRMT5, SLC25A6, PABPC4, PSTPIP2) using RNA sequencing, RNA immunoprecipitation sequencing, and protein interaction profiling. A prognostic model was constructed using univariate Cox regression and least absolute shrinkage and selection operator (LASSO) regression in the GSE10846 dataset and validated in two independent cohorts. DLBCL patients were stratified into high- and low-risk groups based on the model, and clinical characteristics were compared. The tumor immune microenvironment (TIME) was analyzed using CIBERSORT and xCell algorithms to explore correlations with the ERG score. Drug sensitivity assays in DLBCL cell lines were performed to validate the model’s predictive capacity for chemotherapy response. Furthermore, the functional role of PABPC4, a key gene in the scoring system, was investigated through *in vitro* and *in vivo* experiments.

**Results:**

A prognostic model including 11 hub genes was established. Patients in the high-risk group exhibited worse clinical outcomes and an immunosuppressive TIME, characterized by altered expression of immune checkpoint-related proteins. This group demonstrated increased sensitivity to vincristine, etoposide, and oxaliplatin. Knockdown of PABPC4 significantly inhibited cell proliferation, reduced colony formation, and delayed tumor growth *in vivo*.

**Conclusions:**

The ERG scoring system offers a robust and precise tool for predicting survival and guiding personalized treatment in DLBCL patients.

## 1 Introduction

Non-Hodgkin lymphoma (NHL) is a common malignant hematological disease, with diffuse large B-cell lymphoma (DLBCL) being the major subtype, accounting for approximately 30–50% of NHL cases (1). DLBCL is often highly aggressive and is characterized by the diffuse growth of medium- to large B-lymphoid cells. On the basis of its cell of origin, DLBCL can be classified into two subtypes: germinal center B-cell-like (GCB) and activated B-cell-like (ABC). These two subtypes have distinct genomic profiles and different clinical outcomes, with the ABC subtype being associated with poorer prognosis (2). The standard chemotherapy regimen for DLBCL is R-CHOP (rituximab, cyclophosphamide, doxorubicin, vincristine, and prednisone). Although this regimen has good safety, 40–50% of patients still experience drug resistance or relapse (3), underscoring the urgent need for robust prognostic biomarkers to guide risk stratification and personalized therapeutic strategies. Recent advances in multi-omics analyses have highlighted the critical role of dysregulated RNA metabolism and protein translation in cancer progression, yet the prognostic implications of these pathways in DLBCL remain underexplored.

ENO1 (alpha-enolase) is a multifunctional protein that catalyzes glycolysis by converting 2-phospho-D-glycerate to phosphoenolpyruvate and enhances cell migration through plasminogen recruitment and plasmin activation (4–8). As an RNA-binding protein, ENO1 stabilizes oncogenic mRNAs (e.g., YAP and IRP1) to promote hepatocarcinogenesis (9, 10). Additionally, it modulates immune responses by interacting with immune-related molecules, potentially facilitating tumor immune evasion through microenvironment remodeling (11–15). These pleiotropic roles position ENO1 as a pivotal regulator of cancer metabolism, invasion, and immunosuppression. However, the broader landscape of ENO1-related genes (ERGs) and their collective impact on DLBCL prognosis and tumor biology remain unknown.

In this study, we performed an integrated analysis of ERGs via data from RNAseq, RIPseq, and protein interaction profiles to explore their functions in DLBCL. We then constructed and validated a risk assessment model that effectively predicts the prognosis of patients with DLBCL. With this model, we identified chemotherapy drugs that are more sensitive to high-risk patients and validated these findings through cell experiments. We also evaluated the relationship between the immune microenvironment and the risk model and found that the high-risk group tended to develop an immunosuppressive TME. Moreover, we are the first to evaluate the impact of the PABPC4 protein on the proliferation and prognosis of DLBCL. Both *in vitro* and *in vivo* experiments demonstrated that high expression of PABPC4 promotes the proliferation of DLBCL and is negatively correlated with overall survival (OS). Our study provides new insights into the role of the ENO1 interaction network in the development of DLBCL and offers guidance for the prognosis and precision medicine of DLBCL patients.

## 2 Materials and methods

The flowchart of this study is shown in **Supplementary Figure 1**. Initially, we performed unsupervised clustering, functional enrichment, survival analysis, and clinical correlation analysis of ENO1−related genes using the GSE10846 dataset. Subsequently, a clinical prognostic model based on 11 ERGs was constructed through Cox regression, LASSO regression, and Kaplan-Meier analysis. The predictive accuracy of this model was externally validated in the GSE87371 and GSE181063 cohorts, with evaluations covering survival outcome, clinical feature correlation, prognostic stratification, tumor immune microenvironment, and drug sensitivity. Furthermore, cell−based assays were conducted to experimentally verify the model’s drug sensitivity predictions. Finally, the functional role of the key gene PABPC4 in DLBCL was validated through both cellular experiments and mouse models. A detailed description of the experimental procedures is provided below.

### 2.1 Identification of ENO1-related genes

#### 2.1.1 RNA sequencing and differential expression analysis

RNA-seq was performed as previously reported (8). Total RNA was isolated from Burkitt lymphoma Daudi cells and Daudi cells with ENO1 knockdown (shENO1) using TRIzol reagent (Invitrogen). RNA quality control was performed by assessing concentration (Qubit 2.0 Fluorometer, Life Technologies) and integrity (Bioanalyzer 2100, Agilent Technologies). Sequencing libraries were prepared and subjected to paired-end 150-bp sequencing on the Illumina NovaSeq platform (Novogene, China). Raw reads were processed using the DESeq2 pipeline to identify differentially expressed genes (DEGs). Genes with |log2 fold change| > 1 (2-fold change) and an FDR-adjusted p-value <0.05 were considered statistically significant. This analysis revealed 82 DEGs significantly altered upon ENO1 knockdown, which were selected for downstream functional investigation.

#### 2.1.2 RIP-seq analysis of ENO1-bound RNAs

To identify ENO1-associated RNAs, we performed RNA immunoprecipitation sequencing (RIP-seq) in Burkitt lymphoma Daudi cells and Daudi cells stably overexpressing ENO1-Flag (Daudi-ENO1-Flag OE). Cells were lysed under native conditions, and ENO1-RNA complexes were immunoprecipitated using an anti-ENO1 antibody (Abcam, #ab227978). Parallel immunoprecipitation with anti-IgG antibody (Abcam, #ab172730) served as the negative control.

Immunoprecipitated RNAs were isolated, and cDNA libraries were prepared for 150 bp paired-end sequencing on an Illumina HiSeq X Ten platform (AB Life, China). To ensure robust peak identification, we employed three independent analytical methods: Piranha (16) (for peak calling based on read density), CIMS (17) (Crosslink-Induced Mutation Site analysis for precise binding site mapping), and ABLife’s proprietary peak-calling algorithm. Only RNA targets consistently identified by all three methods (n=32) with ≥2-fold enrichment and FDR-adjusted p-value <0.05 were considered high-confidence ENO1-binding partners and selected for downstream analysis.

#### 2.1.3 Protein-protein interaction mass spectrometry analysis

To identify ENO1-interacting proteins, we performed immunoprecipitation coupled with mass spectrometry (IP-MS) in Burkitt lymphoma Raji cells. Cell lysates were pre-cleared with Protein A/G beads and subsequently immunoprecipitated overnight at 4°C using an anti-ENO1 antibody (Abcam, #ab227978), with anti-IgG antibody (Abcam, #ab172730) serving as the negative control. Immune complexes were rigorously washed with low-salt buffer (50 mM Tris-HCl pH 7.4, 150 mM NaCl, 0.1% NP-40) and high-salt buffer (50 mM Tris-HCl pH 7.4, 500 mM NaCl, 0.1% NP-40) (each wash repeated five times to minimize non-specific binding). Bound proteins were eluted and digested with trypsin (Promega) for LC-MS/MS analysis (performed by Novogene, China). MS data were processed using MaxQuant and searched against the UniProt human protein database. High-confidence ENO1-interacting proteins were defined as those showing≥4-fold enrichment (log2FC≥2) in ENO1-IP versus IgG control with an FDR-adjusted p-value<0.05, identifying 345 candidates for subsequent validation and functional studies.

In total, 459 genes were identified as ERGs.

### 2.2 Functional enrichment analysis

GO (Gene Ontology) and KEGG (Kyoto Encyclopedia of Genes and Genomes) enrichment analyses were performed with the “clusterProfiler R” package (18).

### 2.3 Data collection

RNA-seq data and clinical information were obtained from GEO (https://www.ncbi.nlm.nih.gov/geo/). The training set comprised 412 DLBCL samples (GSE10846), while validation used 1,144 (GSE181063) and 221 samples (GSE87371). Gene expression data and corresponding clinical data from each dataset were retrieved using Bioconductor packages (19). Ethical approval for public database use was granted by Dalian Medical University’s ethics committee.

### 2.4 Consensus clustering analysis of ERGs

Patients with DLBCL from the GSE10846 cohort were clustered into distinct subtypes via the “ConsensusClusterPlus” package (20) in R software according to the expression of ERGs. OS (overall survival) analysis was performed via KM (Kaplan-Meier) curves in the different clusters. The clinical features of the different clusters were analyzed and are shown in the form of heatmaps.

### 2.5 Construction and validation of the ERG scoring model

Prognostic ERGs were identified using univariate Cox regression (“survival” package) (21) and LASSO analysis (“glmnet” package) (22) in GSE10846. An 11-gene risk signature was derived via multivariate Cox regression, with scores calculated as: ERG score = ∑ (Expi × coefi) (Expi = gene expression; coefi = coefficient). Patients were stratified into low-/high-risk groups by median score.

Next, principal component analysis (PCA) was performed to validate the reliability of clustering on the basis of the ERG score via the “stats” package of R. OS analysis on the basis of the KM curve was performed in different risk groups. Time-dependent receiver operating characteristic (ROC) curve analysis was conducted via the “Time ROC” package (23) in R to assess the accuracy and reliability of the ERG scoring signature.

### 2.6 Clinical correlations and independent prognostic value of the ERG risk score

Clinical feature differences between risk groups were analyzed using Wilcoxon and chi-square tests (GSE10846 dataset). Univariate/multivariate Cox analyses assessed the ERG score’s independent prognostic value. To explore the interrelationship of the different variables, a nomogram was generated via the “rms” package of R.

### 2.7 Immune landscape analysis

The infiltrating immune cell compositions were calculated via CIBERSORT (24) and xCell (25) and compared between the high- and low-risk groups in the GSE10846 dataset. The immune score, stromal score and estimate score were calculated via the ESTIMATE algorithm. The microenvironment score was calculated via xCell.

### 2.8 Evaluation of drug sensitivity

The R package “oncoPredict” (26) was used to predict the half-maximal inhibitory concentration (IC_50_) of chemotherapeutic drugs on the basis of the Genomics of Drug Sensitivity in Cancer (GDSC). The estimated results were compared between the high-risk and low-risk groups.

### 2.9 Quantitative real-time polymerase chain reaction and ERG risk scores for DLBCL cell lines

Total RNA was extracted using TRIzol and reverse transcribed to cDNA. Gene expression levels in 6 DLBCL cell lines were quantified by qRT-PCR (SYBR^®^ Green Premix, Accurate Biotechnology) using the 2^−ΔCt^ method. ERG risk scores were calculated as: ERG score = ∑ (Expi × coefi). Primer sequences are provided in **Supplementary Materials**.

### 2.10 Cell proliferation assay

The cells were seeded in 96-well plates at a density of 5,000 cells/well in complete medium. After incubation with drugs at different concentrations for 48 h, CCK8 reagent was added to each well, and the cells were incubated in a cell incubator for 2 h. The absorbance was measured at 450 nm via a microplate reader (Ex800; Biotek).

### 2.11 Stable cell line construction

sgRNAs were inserted into the LentiCRISPRv2 plasmid according to the manufacturer’s instructions. Lentivirus packaging was performed as previously reported (8). The cells were subsequently transfected with lentivirus. Polybrene was used to increase transduction efficiency. Stable cell lines were obtained via puromycin selection.

The sequences of the sgRNAs are listed below.

sgPABPC4-1: 5’- caccGCAGCCACTCGTTGCATATAC-3’

sgPABPC4-2: 5’- caccGCAACCAGTATATGCAACGAG-3’

### 2.12 Colony formation assay

Cells were seeded into 24-well plates with 1,000 cells per well and cultured in complete medium supplemented with 1.3% methylcellulose (Sigma). The colonies were photographed via a microscope (Olympus) after 10 days.

### 2.13 *In vivo* experiments

The *in vivo* study procedures were conducted in accordance with the guidelines of the Institutional Animal Care and Use Committee and approved by the Institutional Ethics Committee of Dalian Medical University (Approval number: AEE20061). We have adhered to ARRIVE guidelines and upload a completed checklist.

Female BALB/c nude mice (aged 4–5 weeks; weight, 14–17 g) were purchased from GemPharmatech Co., Ltd. (Nanjing, China) and housed in a specific pathogen-free (SPF) facility under controlled conditions in Dalian Medical University. Mice were randomly allocated using stratified randomization based on body weight. To assess the impact of PABPC4 on the proliferation of tumor cells *in vivo*, we employed the method of subcutaneous tumorigenesis. Pilot studies indicated a 50% tumor formation rate for SU-DHL4 cells in BALB/c nude mice. To ensure statistical power with anticipated attrition, we established xenografts in 10 mice per group (allowing detection of ≥2-fold differences with 80% power at α=0.05, based on two-tailed t-test assumptions). Control SU-DHL4 cells (1×10^7^ in 100 μL Matrigel) were subcutaneously injected into the right anterior flank, while SU-DHL4-sgPABPC4 cells (1×10^7^ in 100 μL Matrigel) were similarly implanted in the contralateral hind limb. Tumor cell injections were performed by a researcher blinded to group identity using coded syringes. The body weights of the mice were measured every other day. Animals failing to develop palpable tumors by Day 20 were excluded from efficacy analysis. Once tumor formation was observed, the size of the tumors was monitored daily. When the tumors reached a size of 20 mm, the mice were euthanized by intraperitoneal injection of pentobarbital sodium (100 mg/kg; Sigma-Aldrich), in accordance with the AVMA Guidelines for Euthanasia (2020). The tumors were then excised, weighed, and then subjected to immunohistochemistry.

### 2.14 Statistical analysis

Statistical analyses used R 4.3.1. Group comparisons employed Wilcoxon or Kruskal-Wallis tests. Cox regression assessed ERG score’s prognostic value. Error bars show SD; significance levels: *p<0.05, **p<0.01, ***p<0.001.

## 3 Results

### 3.1 Identification of ERGs and functional enrichment analysis

**Figure 1:**
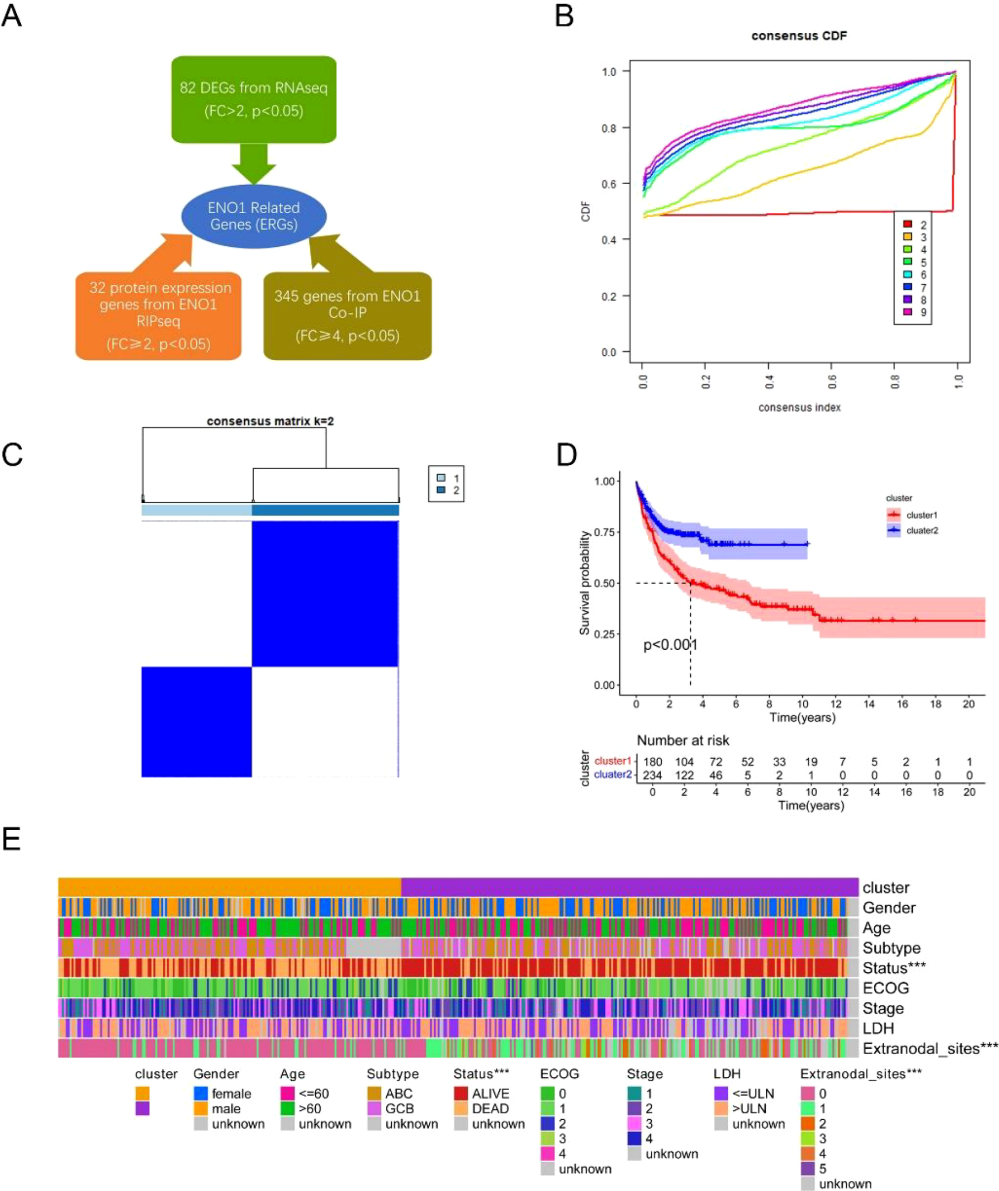
Identification of ENO1-related genes (ERGs) and consensus clustering analysis of identified ERGs in the GSE10846 dataset. **(A)** Differentially expressed genes between the Daudi and Daudi-shENO1 groups, protein expression genes from ENO1 RIPseq, and ENO1-interacting protein genes from Co-IP were selected as ERGs. **(B)** Consensus matrix heatmap defining two clusters (k = 2). **(C)** Consensus clustering cumulative distribution function (CDF) with k values ranging from 2–9 in the GSE10846 dataset. **(D)** KM curve for the two clusters. **(E)** Differences in clinical characteristics between the two distinct clusters.

Integrated analysis of transcriptomic, RIP-seq, and PPI-MS data identified 459 ERGs (**Figure 1A**). GO/KEGG analyses revealed enrichment in RNA splicing, translation initiation, and mRNA metabolic processes (**Supplementary Figures 2A, B**), suggesting ERGs’ role in RNA processing and protein synthesis.

### 3.2 Molecular clustering of the ERGs in DLBCL

Given the low incidence rate of Burkitt lymphoma (BL) (27), which poses challenges for building clinical prediction models requiring large sample sizes, and considering that DLBCL shares key clinical features with BL—such as high proliferative and invasive characteristics—while exhibiting a higher prevalence (28), we selected DLBCL for molecular clustering analysis based on ERG expression.

We performed a consensus clustering analysis of the GSE10846 dataset to investigate the relationship between the expression of 459 ERGs and the prognosis of patients with DLBCL. The optimal number of clusters (k = 2) was determined from the CDF curve (**Figure 1B**). The 414 patients with DLBCL could be divided into two clusters (Cluster 1: n=180; Cluster 2: n=234) according to the expression of ERGs (**Figure 1C**). Survival analysis revealed that Cluster 1 was significantly correlated with worse OS than was Cluster 2 (**Figure 1D**). We subsequently compared the clinical features between the two clusters. The heatmap revealed that Cluster 2 was significantly correlated with the number of extranodal sites (**Figure 1E**).

### 3.3 Construction of a prognostic ERG scoring model

**Figure 2:**
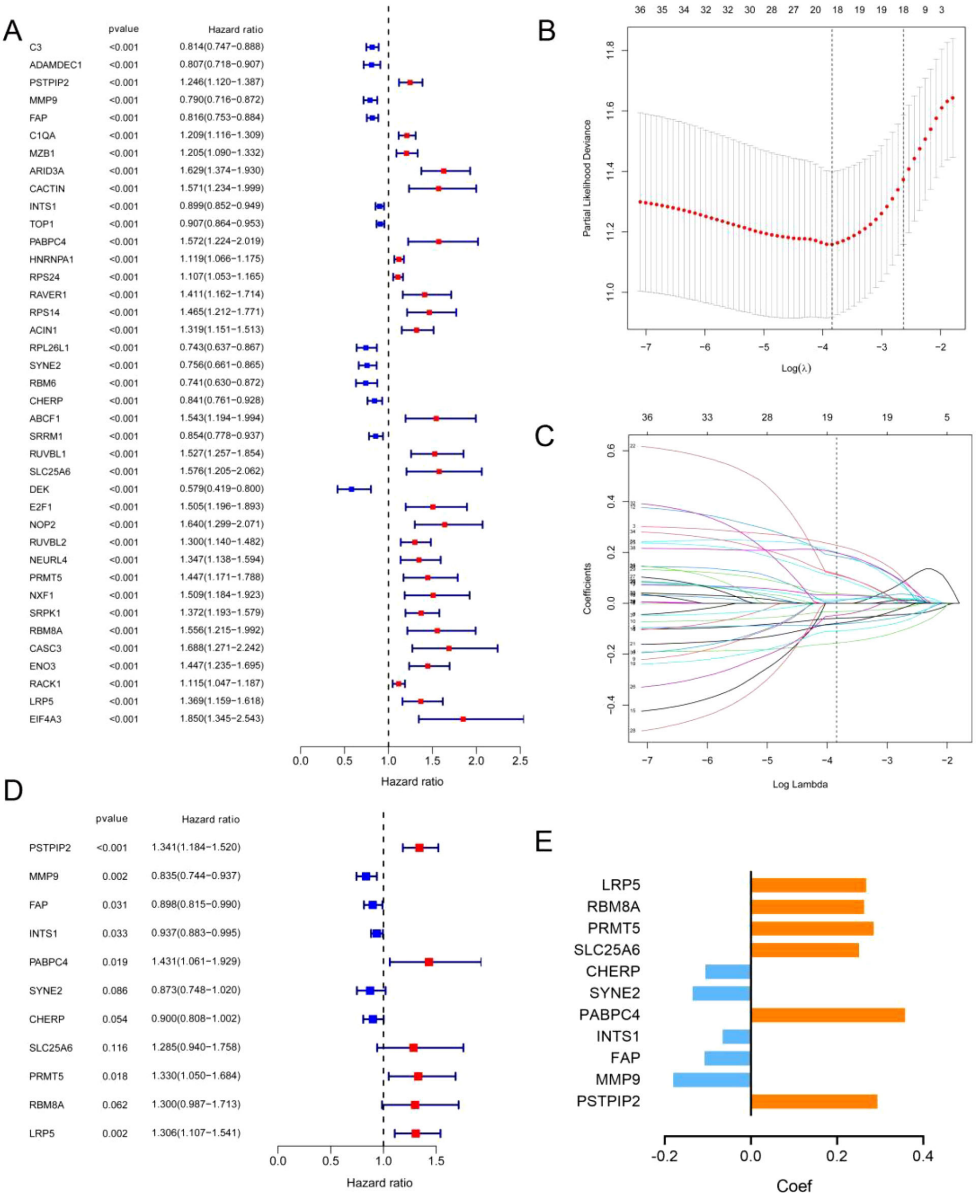
Identification of prognostic ERGs in the GSE10846 dataset. **(A)** P values and hazard ratios of 39 ERGs related to DLBCL prognosis according to univariate Cox regression analysis. **(B)** Selection of the optimal parameter (lambda) in the LASSO model. **(C)** LASSO coefficient profiles of 39 ERGs from univariate Cox regression analysis. **(D)** P values and hazard ratios of the 11 retained candidate genes according to multivariate Cox regression analysis. **(E)** Multivariate Cox coefficient of the 11 candidate genes for ERG scoring model construction.

To obtain a more applicable and reliable classifier to predict the prognosis of patients with DLBCL, univariate Cox regression was used, and 39 prognosis-related genes (p<0.001) were identified (**Figure 2A**). GO and KEGG enrichment analyses revealed that these genes were closely related to RNA splicing, the spliceosomal complex, the exon–exon junction complex, ATP hydrolysis activity, and helicase activity (**Supplementary Figures 2C, D**). LASSO/multivariate Cox analyses yielded an 11-gene signature: protective genes (CHERP, SYNE2, INTS1, FAP, MMP9; HR<1) and risk genes (LRP5, RBM8A, PRMT5, SLC25A6, PABPC4, PSTPIP2; HR>1) (**Figures 2B–E**, **Supplementary Figure 3**).

Among the 11 ERG genes, PSTPIP2, MMP9, and FAP were identified from RNA-seq as transcriptionally regulated by ENO1, showing significant downregulation upon shENO1 (logFC = -1.5, -1.6, -1.2; all *p* < 0.05 vs. controls); RIP-seq confirmed direct binding of ENO1 protein to INTS1 mRNA (5-fold enrichment vs. IgG, *p* < 0.05); and PPI-MS revealed ENO1-centric protein complexes, including strong binding to PABPC4 (6.8-fold vs. IgG, *p* < 0.001) and exclusive interactions with CHERP, SYNE2, LRP5, RBM8A, PRMT5, and SLC25A6 (undetected in IgG controls; **Supplementary Table 1**).

Network analysis (GeneMANIA, https://genemania.org; **Supplementary Figure 4**) demonstrated significant co-expression and genetic interactions among these genes, with functional annotation implicating mRNA splicing, RNA 3’-end processing, and coagulation pathways. Strikingly, subgroup enrichment analysis (DAVID, https://davidbioinformatics.nih.gov/summary.jsp; **Supplementary Table 2**) revealed mechanistic convergence. The RNA-binding module (ENO1, RBM8A, PABPC4, CHERP) was enriched in RNA stability/degradation and mRNA surveillance pathways (post-transcriptional regulation). And the protease module (FAP, MMP9) participated in endopeptidase activity, linking to extracellular matrix remodeling and metastasis. These data collectively validate the 11 ERGs as functionally coordinated partners of ENO1 in DLBCL pathogenesis.

**Figure 3:**
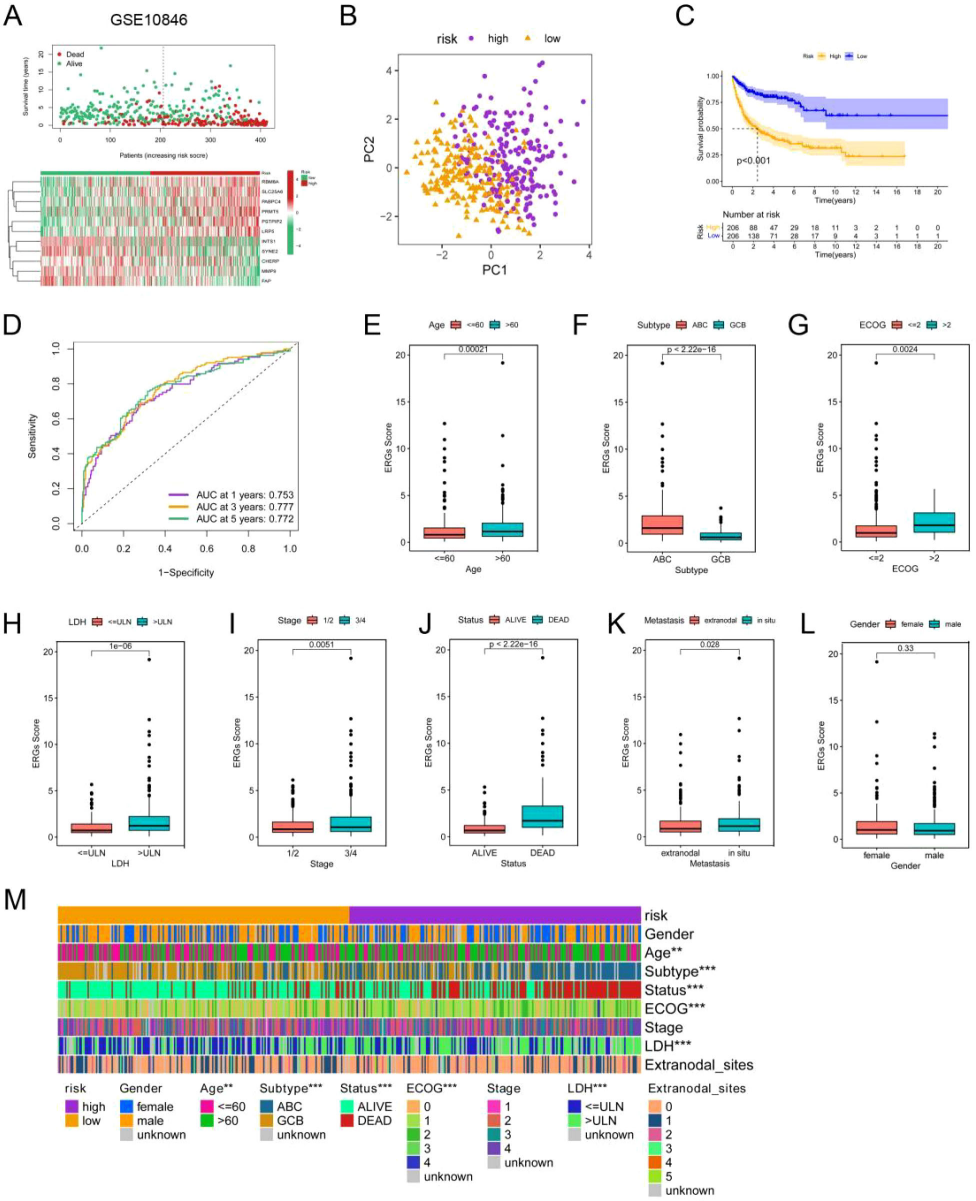
Construction and validation of the ERG scoring model and correlations between the ERG score and clinical features in the GSE10864 dataset. **(A)** Distribution of the survival status and ERG expression in DLBCL patients in the GSE10864 cohorts. **(B)** PCA of the DLBCL patients in the GSE10864 cohorts on the basis of the ERG score. **(C)** KM analyses of the ERG scores in theGSE10864 cohorts. **(D)** Time-dependent ROC curves of the ERG scores in the GSE10864 cohorts. Correlations of the ERG score with age **(E)**, subtype **(F)**, ECOG score **(G)**, LDH level **(H)**, clinical stage **(I)**, patient status **(J)**, extranodal infiltration **(K)**, and gender **(L)**. **(M)** Heatmap showing the differences in clinical characteristics between the two risk clusters. *p< 0.05; **p< 0.01; ***p< 0.001.

Next, the risk score of each DLBCL patient in GSE10846 was calculated using the gene expression level and the estimated coefficient according to the formula mentioned in the methods section. Patients were then divided into high-risk and low-risk groups according to the median risk score (**Supplementary Figure 5A**). The scatter plot revealed a greater mortality rate in the high-risk group than in the low-risk group (**Figure 3A**). DLBCL patients were well separated into two clusters after PCA (**Figure 3B**). The KM curve further indicated a worse clinical outcome in the high-risk group (**Figure 3C**). Finally, we evaluated the model via time-dependent ROC analysis. The areas under the curve (AUCs) for 1-, 3-, and 5-year OS were 0.753, 0.777, and 0.772, respectively (**Figure 3D**). These results indicated good prediction performance of the ERG scoring model in DLBCL patients.

### 3.4 Validation of the ERG scoring model

To evaluate the predictive performance of the ERG scoring model, datasets GSE181063 and GSE87371 were used as validation sets. Each patient in the datasets was calculated for risk score via the formula obtained previously and then divided into high-risk and low-risk groups on the basis of the median score, with red representing the high-risk group and green representing the low-risk group (**Supplementary Figures 5B, C**). The high-risk group had a higher mortality rate (**Supplementary Figures 5D, E**). Similarly, the PCA results revealed that the high-risk and low-risk groups were distinctly separated into two clusters (**Supplementary Figures 5F, G**), with the high-risk group having a poorer prognosis (**Supplementary Figures 5H, I**). Finally, the area under the curve (AUC) for the GSE181063 dataset at 1, 3, and 5 years was 0.626, 0.612, and 0.603, respectively; for the GSE87371 dataset, the AUCs at 1, 3, and 5 years were 0.661, 0.679, and 0.714, respectively (**Supplementary Figures 5J, K**). These results indicated that the ERG scoring model had excellent performance in predicting the prognosis of DLBCL patients.

### 3.5 Analysis of the correlations between ERG scores and clinical features

Next, we analyzed the correlation between the ERG score and the clinicopathological characteristics of DLBCL patients in the GSE10846 dataset. We observed that ERG risk scores were higher in patients over the age of 60, patients with the ABC subtype, patients with an Eastern Cooperative Oncology Group (ECOG) score greater than 2, and patients with serum LDH concentrations above the normal value. Additionally, the ERG risk score was significantly associated with clinical stage and metastasis status, but it was not significantly related to sex (**Figures 3E–M**). These results were also validated in the GSE181063 and GSE87371 datasets (**Supplementary Figures 6, 7**).

**Figure 4:**
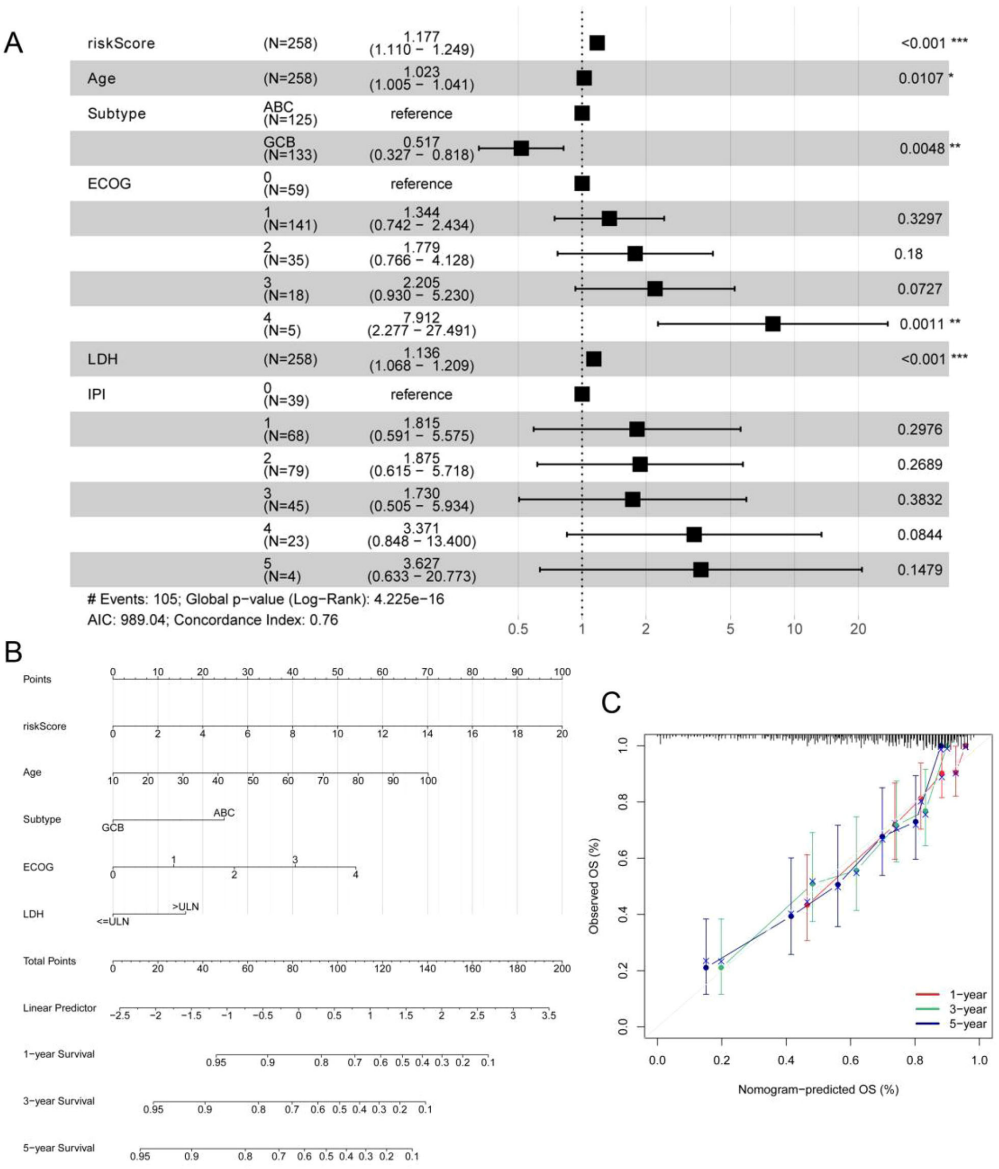
Multivariate Cox regression analysis and the nomogram based on the ERG score, IPI and clinical features in the GSE10846 cohort. **(A)** Multivariate Cox regression analysis of ERG score, IPI and clinical features in GSE10846 training cohort. **(B)** A nomogram for the prediction of the 1-, 3- and 5-year survival probabilities of DLBCL patients according to the ERG score, IPI and clinical factors. **(C)** Nomogram-predicted percentages and the observed probabilities of 1-, 3- and 5-year survival. *p< 0.05; **p< 0.01; ***p< 0.001.

To further assess whether the ERG scoring model is an independent prognostic factor for predicting OS in DLBCL patients, we conducted univariate and multivariate regression analyses in the GSE10846 dataset. The results of the univariate regression analysis revealed that the ERG score, age, subtype, Eastern Cooperative Oncology Group (ECOG) score, and LDH were significantly associated with the survival of DLBCL patients (**Supplementary Table 3**). The results of the multivariate regression analysis indicated that the ERG score is an independent prognostic factor for evaluating the OS of DLBCL patients, with HR = 1.17, 95% CI = 1.10–1.24, and p < 0.001 (**Figure 4A**). To further evaluate the clinical utility of the model, we constructed a prognostic nomogram based on the ERG score and clinical features (**Figure 4B**). The calibration curve results demonstrated that the model had good prognostic ability (**Figure 4C**).

### 3.6 Correlation between the ERG risk score and the immune landscape

**Figure 5:**
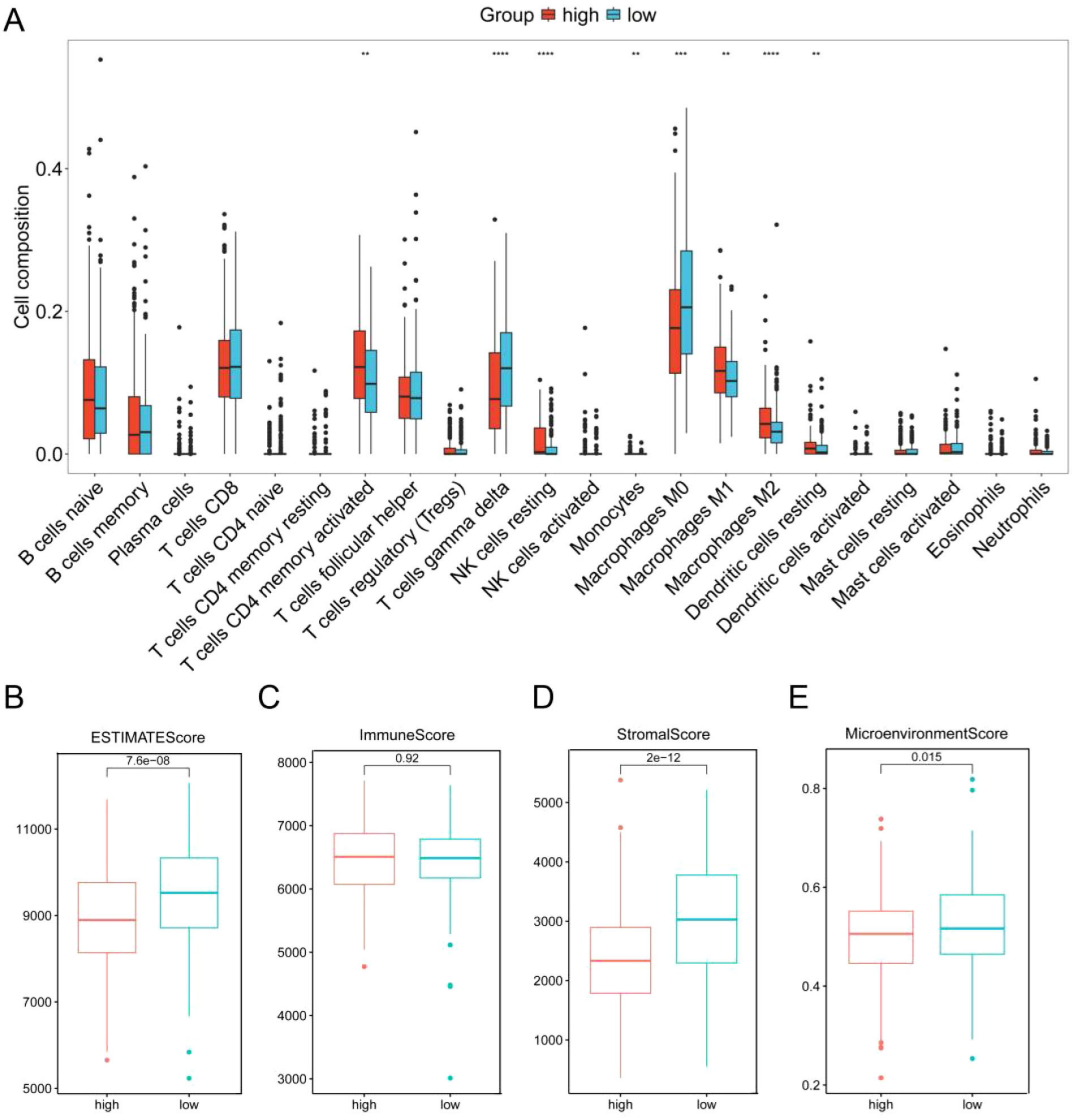
Correlation analysis of the ERG score with the immune landscape in GSE10846. **(A)** Proportion of 22 immune cells between the low-risk and high-risk groups according to CIBERSORT. ESTIMATE score **(B)**, stromal score **(C)**, immune score **(D)** and microenvironment score **(E)** between the high-score and low-score groups. **p< 0.01; ***p< 0.001; ****p< 0.0001.

We next investigated the potential correlation between the ERG score and the immune landscape of DLBCL. Analysis using the CIBERSORT algorithm revealed a distinct immune profile in the high-risk group, characterized by significantly reduced levels of gamma delta T cells and M0 macrophages, alongside significantly elevated levels of resting NK cells and M2 macrophages compared to the low-risk group (**Figure 5A**). This shift is biologically significant because gamma delta T cells are known for their potent innate anti-tumor activity, including direct cytotoxicity and cytokine production (29). Conversely, M2 macrophages are classically associated with promoting tumor progression by suppressing immune responses, facilitating angiogenesis, and supporting tissue remodeling in the tumor microenvironment (TME) (30). The observed decrease in potentially pro-inflammatory M0 macrophages and increase in immunosuppressive M2 macrophages further supports an immunosuppressive shift. The increase in resting NK cells (which typically have lower cytotoxic activity than activated NK cells) may also contribute to diminished immune surveillance.

Further supporting this interpretation, analysis via the xCell algorithm (**Supplementary Figure 8**) confirmed the significant decrease in CD8^+^ T cells and additionally revealed significant increases in activated dendritic cells (aDCs), memory B cells, NKT cells, and Th1 cells within the high-risk group. While the increase in Th1 cells and NKT cells might initially suggest enhanced anti-tumor immunity, the overall context is crucial. The significant loss of key cytotoxic effectors (gamma delta T cells, CD8^+^ T cells), coupled with the dominance of immunosuppressive M2 macrophages, suggests that any potential pro-inflammatory signals from Th1/NKT cells may be insufficient or actively suppressed within this TME. The role of increased aDCs and memory B cells in this specific high-risk context warrants further investigation but does not negate the strong immunosuppressive signals from the other observed changes.

Critically, the collective impact of these cellular alterations points towards an immunosuppressed TME in the high-risk group. This conclusion is further substantiated by results from the ESTIMATE algorithm. The high-risk group exhibited significantly lower ESTIMATE scores, stromal scores, and microenvironment scores compared to the low-risk group (**Figures 5B–E**). These lower scores strongly imply a reduction in the overall immune cell infiltration and stromal components within the TME of high-risk patients. This depletion of immune cells aligns with the observed decrease in key anti-tumor effectors and reinforces the notion of an immune-evasive microenvironment.

Further characterizing the immunosuppressive TME, we observed significant dysregulation of immune checkpoint molecules in the high-risk group (**Supplementary Figure 9A**). Critically, there was significant downregulation of genes associated with immune activation and co-stimulation, including B2M, CD28, CD40LG, ICOS, CD86, IL23A, and LDHA. The downregulation of these molecules likely impairs T cell activation, antigen presentation, and effector function (31–34). Conversely, genes associated with immune suppression or exhaustion were significantly upregulated, including LGALS9 [galectin-9, ligand for TIM-3 (35)], TNFSF9 [4-1BBL, complex role but often associated with exhaustion in chronic settings (36)], YTHDF1 [linked to immunosuppression (37)], and PVR [CD155, ligand for inhibitory receptors TIGIT/CD96 (38)]. Strikingly, the downregulation of activating/co-stimulatory genes correlated with poor prognosis, while upregulation of inhibitory/exhaustion-related genes was associated with worse clinical outcomes (**Supplementary Figure 9B**).

Therefore, our integrated analyses demonstrate that a high ERG risk score is associated with an immune landscape characterized by immunosuppressive polarization and dysregulated immune checkpoints expression, which provides a mechanistic basis linking high ERG scores to poor clinical outcomes in DLBCL.

### 3.7 Prognostic value of the ERG score in the prediction of therapeutic response

**Figure 6:**
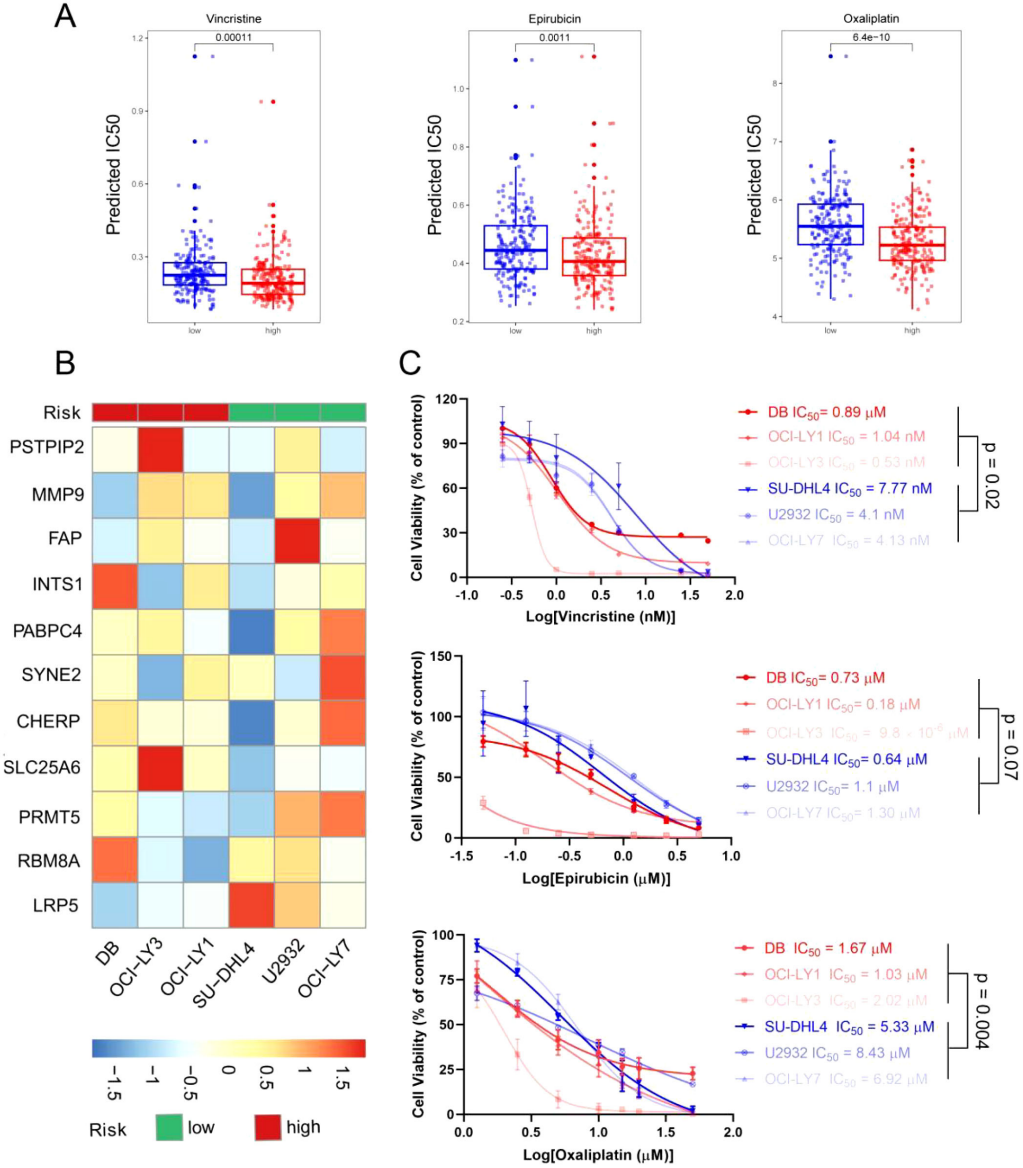
Evaluation of therapeutic response and drug sensitivity via the ERG scoring model. **(A)** The predicted IC_50_ values of vincristine, epirubicin, and oxaliplatin between the high- and low-risk groups via the R package “oncoPredict”. **(B)** Six DLBCL cell lines were separated into high- and low-risk groups on the basis of the ERG scoring model. **(C)** The actual IC_50_ values of vincristine, epirubicin, and oxaliplatin in the 6 DLBCL cell lines.

Although drug resistance is a challenge in the treatment of DLBCL, the R-CHOP regimen remains the first-line chemotherapy regimen for DLBCL in clinical practice. Studies have shown that providing patients with personalized treatment plans can improve the remission rate of the disease. Therefore, we further conducted an analysis of the correlation between ERG scores and the sensitivity of patients to chemotherapy drugs via the “oncoPredict” R package. We found that patients in the high-risk group were more sensitive to vincristine (p < 0.001), etoposide (p = 0.001), and platinum drugs (p < 0.001) (**Figure 6A**).

To verify the accuracy of this prediction, we conducted further validation in DLBCL cell lines. First, we detected the expression levels of the 11 prognostic genes via qRT–PCR (**Supplementary Table 4**) and divided the 6 DLBCL cell lines into high- and low-risk groups on the basis of the previous risk scoring formula. Among them, OCI-LY1, OCI-LY3, and DB were in the high-risk group, whereas OCI-LY7, U2932, and SU-DHL4 were in the low-risk group (**Figure 6B**). Next, we detected the IC_50_ values of vincristine, epirubicin, and oxaliplatin in 6 cell lines via a cell viability assay. The results are shown in **Figure 6C**, where high-risk group cells were more sensitive to vincristine and oxaliplatin, with p values of 0.02 and 0.004, respectively. The difference in the sensitivity of high- and low-risk cells to epirubicin was not significant. However, the epirubicin IC_50_ values of the high-risk group cells were lower than those of the low-risk group cells. These results were consistent with the predictions from bioinformatics, suggesting that the sensitivity of DLBCL patients to chemotherapy drugs can be predicted on the basis of ERG scores.

### 3.8 Experimental evaluation of PABPC4 function in DLBCL

In our constructed ERG scoring system, we noted that PABPC4 contributes the most to the degree of risk. To date, the role of PABPC4 in the development of DLBCL has not been reported. Therefore, we first analyzed the expression of PABPC4 in DLBCL via the GEPIA website (http://gepia.cancer-pku.cn). The results revealed that PABPC4 was significantly upregulated in DLBCL (**Supplementary Figure 10A**). We then analyzed the relationship between PABPC4 expression and the OS of DLBCL patients in the GSE10846 dataset. We found that patients with high expression of PABPC4 had a poorer prognosis (**Supplementary Figure 10B**). These results suggest that PABPC4 may promote the occurrence of DLBCL.

**Figure 7:**
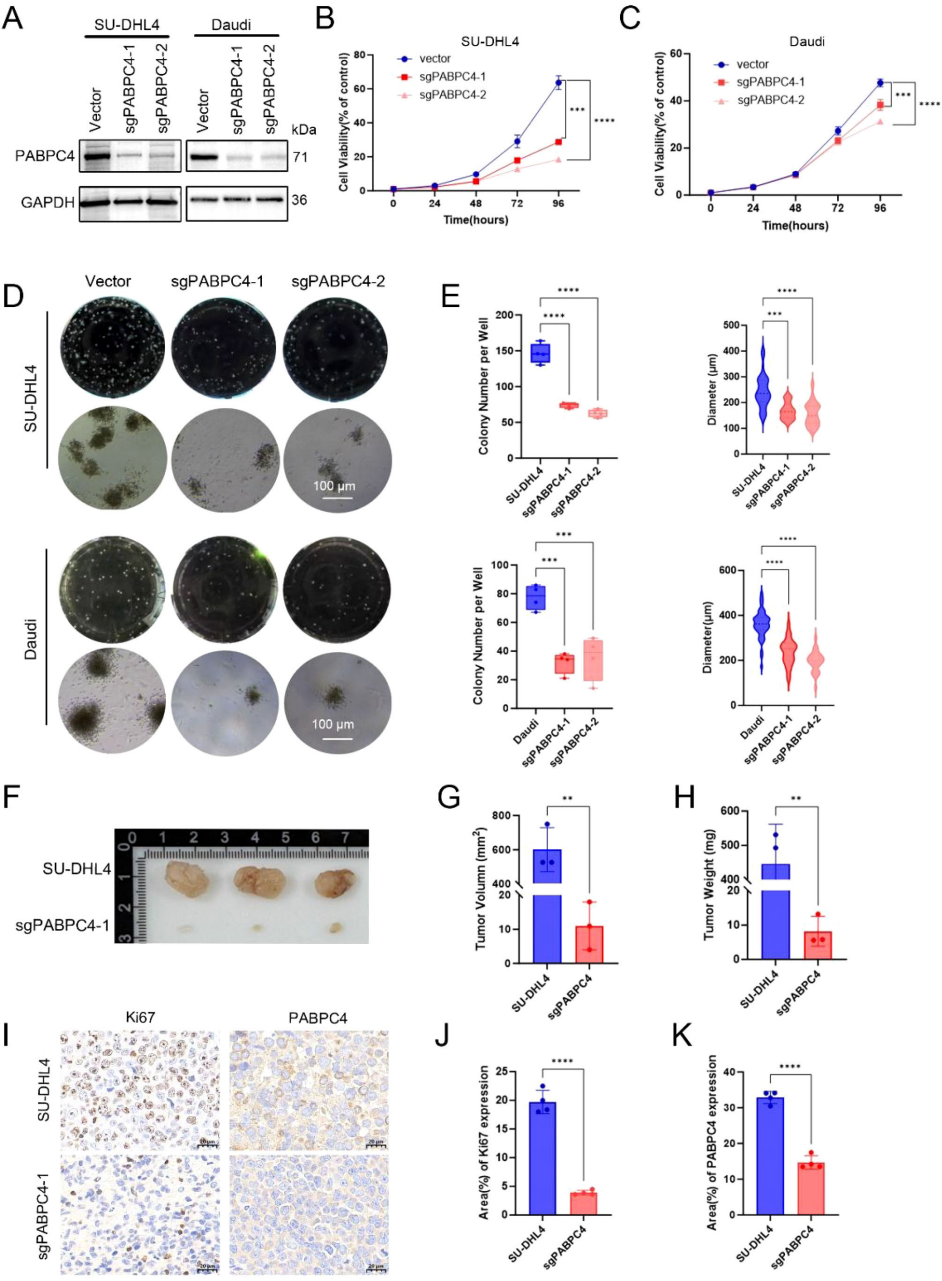
Knockdown of PABPC4 inhibits B lymphoma cell proliferation and colony formation *in vitro* and *in vivo*. **(A)** PABPC4 expression in stable sgPABPC4 SU-DHL4 and Daudi cell lines. **(B, C)** Cell viability of control and sgPABPC4 cells at different time points. **(D)** Effects of PABPC4 knockdown on cell colony formation. Representative images of colony formation by different cells are shown. **(E)** Statistical analysis of the colony numbers and colony diameters in **(D)**. Representative images **(F)**, tumor volumes **(G)**, and tumor weights **(H)** of xenografts derived from the indicated cells are shown. **(I)** Immunohistochemical results of PABPC4 and Ki67 expression in tumors from different xenograft groups. **(J, K)** Statistical analysis of the results in **(I)**. **p< 0.01; ***p< 0.001; ****p< 0.0001.

Next, we constructed PABPC4-knockdown cell lines from the DLBCL cell line SU-DHL4 and the Burkitt lymphoma cell line Daudi (**Figure 7A**). Compared with that of control cells, the proliferation rate of sgPABPC4 SU-DHL4 cells was reduced by approximately 50% (**Figures 7B, C**). Moreover, the number of colonies formed by sgPABPC4 cells was approximately 60% of that formed by control cells. The viability and number of colonies formed by Daudi-sgPABPC4 cells were approximately 30% and 50% of those formed by the control cells, respectively. (**Figures 7D, E**). Furthermore, we assessed the impact of PABPC4 on the proliferation of DLBCL tumors *in vivo* via the subcutaneous tumorigenesis method. Compared with that of the control group, the proliferation ability of sgPABPC4 cells *in vivo* was significantly reduced (**Figures 7F–H**), while no significant change in the body weight of the mice was observed (**Supplementary Figure 10C**). Immunohistochemical results revealed that the Ki67 index percentage in the sgPABPC4 group was significantly lower than that in the control group (**Figures 7I–K**). These results indicate that PABPC4 can promote the proliferation of DLBCL cells both *in vitro* and *in vivo*.

## 4 Discussion

The pronounced heterogeneity in survival outcomes among DLBCL patients underscores the critical need for refined risk stratification. While traditional clinical indices (IPI, R-IPI, NCCN-IPI) offer valuable prognostic guidance, their reliance solely on clinical parameters overlooks the impact of molecular heterogeneity and tumor microenvironment (TME) dynamics (39, 40). Our study addressed this gap by integrating gene expression profiles of ERGs with clinical variables. In this study, we established a prognostic evaluation model based on ERGs via machine learning methods. This ERG-based classifier assessed the prognosis of DLBCL patients effectively and emerged as an independent prognostic factor in multivariate analysis. Critically, the model’s alignment with adverse clinical features (including age >60 years, ABC subtype, advanced stage, and elevated LDH) reinforces its biological relevance and potential utility in treatment personalization.

Beyond prognostication, our model reveals fundamental mechanisms of DLBCL progression through tumor microenvironment (TME) reprogramming. High-risk patients exhibited profound immunosuppressive remodeling characterized by two synergistic alterations: (1) Cellular imbalance featuring enrichment of pro-tumorigenic M2 macrophages (established promoters of DLBCL malignant phenotypes (41, 42)) and depletion of CD8^+^ T cells [critical effectors of antitumor immunity (43, 44)]; and (2) Immune checkpoint dysregulation marked by elevated LAG3 [an inhibitory receptor that suppresses T-cell activation via MHC class II binding (45, 46)] coupled with suppression of B2M [impairing MHC-I antigen presentation (31)] and CD28 [compromising T-cell co-stimulation (48)]. These findings illuminate how ERG-driven molecular programs foster an immune-tolerant niche, potentially explaining the poor outcomes in high-risk patients and suggesting actionable targets for immunotherapy.

Precision medicine can improve treatment outcomes and prolong patient survival. Our model’s capacity to predict therapeutic response represents a key translational advance. We found that patients in the high-risk group were more sensitive to vincristine, epirubicin, and oxaliplatin, which was validated in six DLBCL cell lines. This facilitates rational drug selection for aggressive DLBCL subsets, moving beyond empirical chemotherapy assignment. Moreover, the convergence of ERG scores with immune checkpoint expression further supports combinatorial strategies.

Additionally, in the ERG scoring system, PABPC4 received the highest score, suggesting its critical role in the progression of DLBCL. We generated stable PABPC4-knockdown cell lines via the CRISPR-Cas9 system, and both *in vitro* and *in vivo* studies demonstrated that the overexpression of PABPC4 promoted tumor proliferation. PABPC4 is an RNA processing protein that plays a crucial role in enhancing translation and mRNA stability, thereby promoting gene expression. Yufeng Yuan and colleagues reported that PABPC4 contributes to liver cancer progression by stabilizing the mRNAs of TRIM37 and CDC27 (47). Our study pioneers the functional characterization of PABPC4 in DLBCL. We had validated that there was an interaction between ENO1 and PABPC4 by co-immunoprecipitation (data not shown), further research is needed to elucidate the mechanistic basis of PABPC4-driven lymphomagenesis, particularly its RNA-stabilizing functions in DLBCL-specific contexts.

More importantly, in this study, we discovered that the functional enrichment of ERGs was associated primarily with signaling pathways related to RNA splicing and RNA stability. Previous studies have shown that ENO1 promotes the development of liver cancer by binding to the YAP and IRP 1 mRNAs (9, 10). However, to date, there have been no reports regarding the involvement of ENO1 in RNA splicing. Therefore, further experimental validation is needed.

Our study has limitations that warrant mention. First, the ERG signature was derived from a BL cell model, which possesses a genetic background distinct from that of DLBCL. Nevertheless, its robust prognostic performance across DLBCL cohorts suggests it captures fundamental biological processes shared among aggressive B-cell lymphomas. Second, the experimental validation does not distinguish whether the interactions between the model genes and ENO1 are direct physical interactions or indirect functional relationships, a question that merits further investigation.

## 5 Conclusion

In conclusion, we developed a scoring model based on ERGs that not only predicts the prognosis of DLBCL patients but also guides therapeutic decision-making. Furthermore, for the first time, we validated both *in vitro* and *in vivo* that PABPC4 promotes the progression of DLBCL, offering new perspectives on the underlying mechanisms of DLBCL development.

## Data Availability

The RNAseq and RIPseq data presented in the study are deposited in the GEO repository, accession number GSE225685 and GSE292371. The mass spectrometry proteomics data have been deposited to the ProteomeXchange Consortium via the iProX partner repository, with the dataset identifier PXD061736.
