# Supplementary material for: ENO1-related gene signature predicts prognosis and therapeutic response in diffuse large B-cell lymphoma
Source: Front Immunol. 2025 Oct 23;16:1644020. doi: 10.3389/fimmu.2025.1644020 (PMC12588913; doi:10.3389/fimmu.2025.1644020)

**Supplementary Materials**

Primers used in this article.

| GAPDH | Forward Primer | CCACTCCTCCACCTTTGAC |
| --- | --- | --- |
|  | Reverse Primer | ACCCTGTTGCTGTAGCCA |
| INTS1 | Forward Primer | CACGACCTGTCCTTCGACAA |
|  | Reverse Primer | AGAAGCTCAGGATCTCGGGT |
| PABPC4 | Forward Primer | GGCCTCCCTACTACCACTCA |
|  | Reverse Primer | CAGCTGTCAGTCAGCCCTTG |
| SYNE2 | Forward Primer | ATTCATGCCTTGCGACCAGA |
|  | Reverse Primer | CCCAGGGGCATCTTTGGAAT |
| CHERP | Forward Primer | CAAGAGTCGAGGGCGTTCTT |
|  | Reverse Primer | GAGCCTTGAGTCAGGGATGG |
| SLC25A6 | Forward Primer | GCAACCTTGCCAACGTCATT |
|  | Reverse Primer | CAGAACTGCGTGTGCTTGTC |
| PRMT5 | Forward Primer | CTGTGGCCAAGCAGGGGTTT |
|  | Reverse Primer | CTCGGAGTTCCTGCGAATCT |
| RBM8A | Forward Primer | AAGCGAAGAAACGGAAGGGT |
|  | Reverse Primer | GCCTGGGCTTCCTTGTATGT |
| LRP5 | Forward Primer | GCTCCCACATCTGTATTGCC |
|  | Reverse Primer | TCCTCGTCGCTCTGGTCAT |
| PSTPIP2 | Forward Primer | CTTCAAGCAGCAAGTAGACA |
|  | Reverse Primer | GTTCACCAGGTTGGCACT |
| MMP9 | Forward Primer | AGGACGGCAATGCTGATG |
|  | Reverse Primer | TCGTAGTTGGCGGTGGTG |
| FAP | Forward Primer | TATTCCATACCCAAAGGC |
|  | Reverse Primer | GACAGGACCGAAACATTC |

**Supplementary Fig Legends**

**Supplementary Fig 1.** The flowchart of this study.

**Supplementary Fig 2.** (A) KEGG analysis of the identified ERGs. (B) GO analysis of the identified ERGs. (C) KEGG and (D) GO analysis of the 39 ERGs from univariate Cox regression analysis.

**Supplementary Fig 3.** **KM analyses the 11 retained candidate genes by Multivariate Cox regression analysis.** KM analyses of genes with negative coefficient value (A) and positive coefficient value (B).

**Supplementary Fig 4.** The networks and functions of the 11 ERG genes were analyzed using GeneMANIA (https://genemania.org).

**Supplementary Fig 5.** The distribution of the ERGs Score of DLBCL patients in high- and low- risk groups in GSE10846 (A), GSE181063 (B) and GSE87371 cohorts (C). Distribution of the survival status and ERG expression of DLBCL patients in the GSE181063 (D) and GSE87371 (E)cohorts. PCA of the DLBCL patients in the GSE181063 (F) and GSE87371 (G) cohorts on the basis of the ERG score. KM analyses of the ERG scores in the GSE181063 (H) and GSE87371 (I) cohorts. Time-dependent ROC curves of the ERG scores in the GSE181063 (J) and GSE87371 cohorts (K).

**Supplementary Fig 6. Correlation between the ERGs Score and clinical features in GSE181063 dataset.** (A) Correlation of the ERGs Score with age, LDH level, patient status, extranodal infiltration, subtype, gender, ECOG score and clinical stage. (B) Heatmap showing the differences in clinical characteristics between the two risk clusters. *p< 0.05; **p< 0.01; ***p< 0.001.

**Supplementary Fig 7.** **Correlation between the ERGs Score and clinical features in GSE87371 dataset.** (A) Correlation of the ERGs Score with age, subtype, gender, clinical stage, and IPI. (B) progression-free survival analyses between the high-score and low-score groups. (C) Heatmap showing the differences in clinical characteristics between the two risk clusters. *p< 0.05; **p< 0.01; ***p< 0.001.

**Supplementary Fig 8.** Correlation analysis of ERGs Score with immune landscape in GSE10846. The proportion of immune cells between low-risk and high-risk groups based on xCell.

**Supplementary Fig 9.** (A) Distribution of 38 immune checkpoints between the high-risk and low-risk groups. (B) KM analyses of the expression of the indicated genes.

**Supplementary Fig 10.** (A) The expression of PABPC4 in DLBCL samples in the TCGA dataset. (B) The correlation between PABPC4 expression and survival in DLBCL patients in the GSE10864 dataset. (C) Body weight changes in tumor-bearing mice. Mice were subcutaneously inoculated with tumors (SU-DHL4 and SU-DHL4-sgPABPC4) on Day 0. Body weight was measured every other day until tumors reached the ethical endpoint size (20 mm diameter). Data are presented as mean ± SEM; n = 10 per group). Statistical significance between groups is indicated (ns, not significant).

**Supplementary Figs**

**Supplementary Fig 1.**


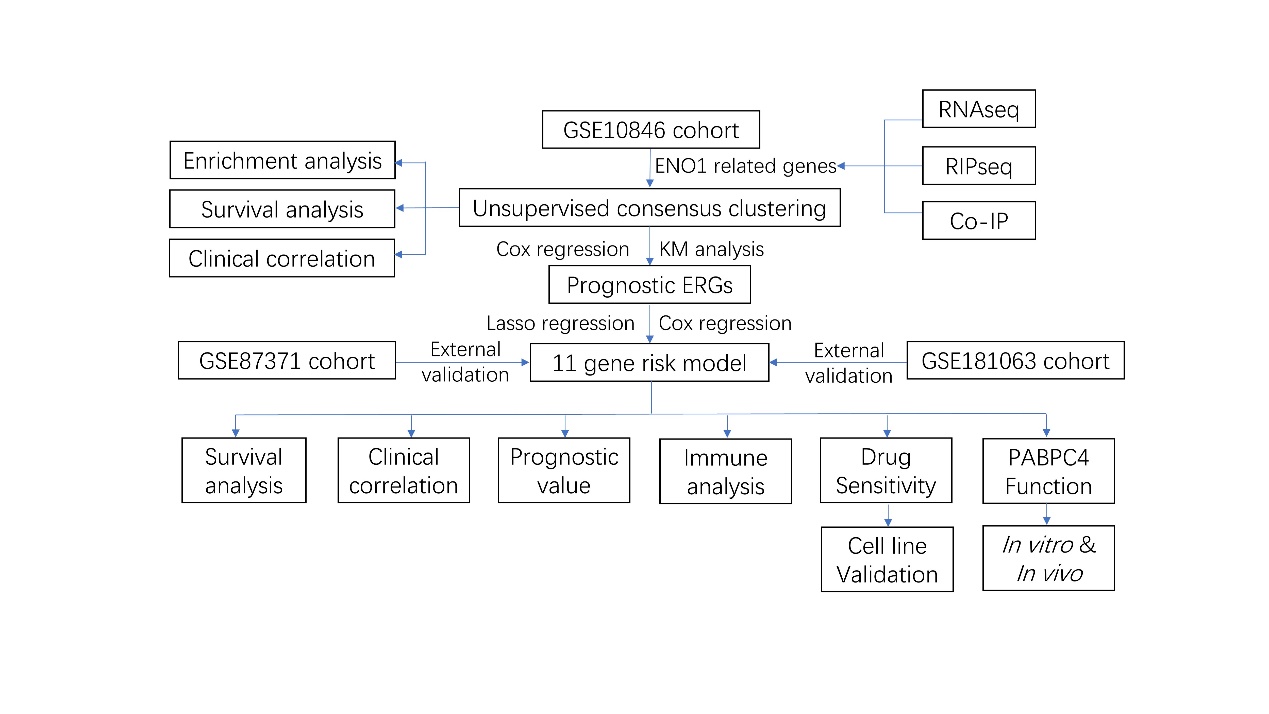


**Supplementary Fig 2.**

**
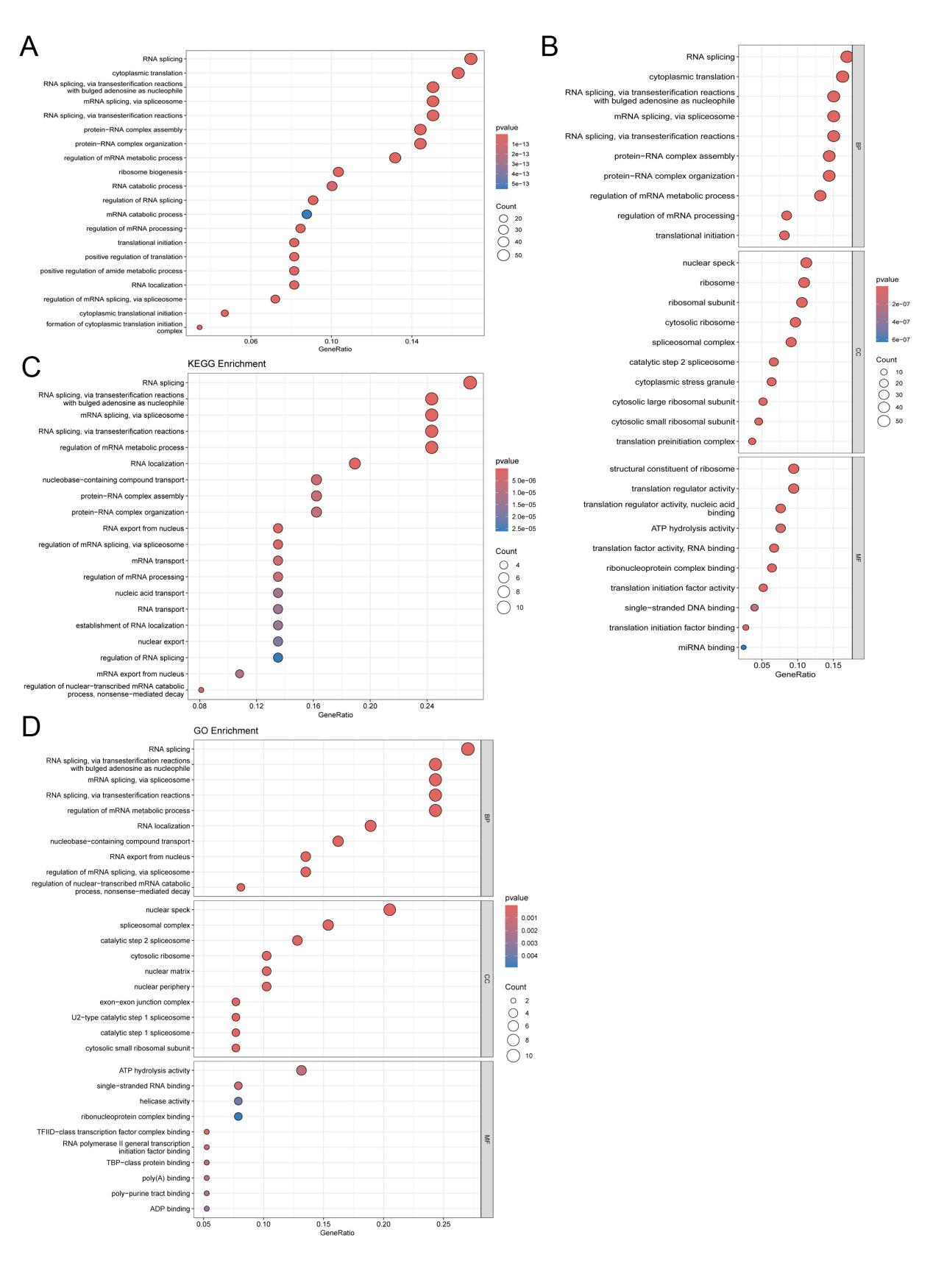
**

**Supplementary Fig 3.**


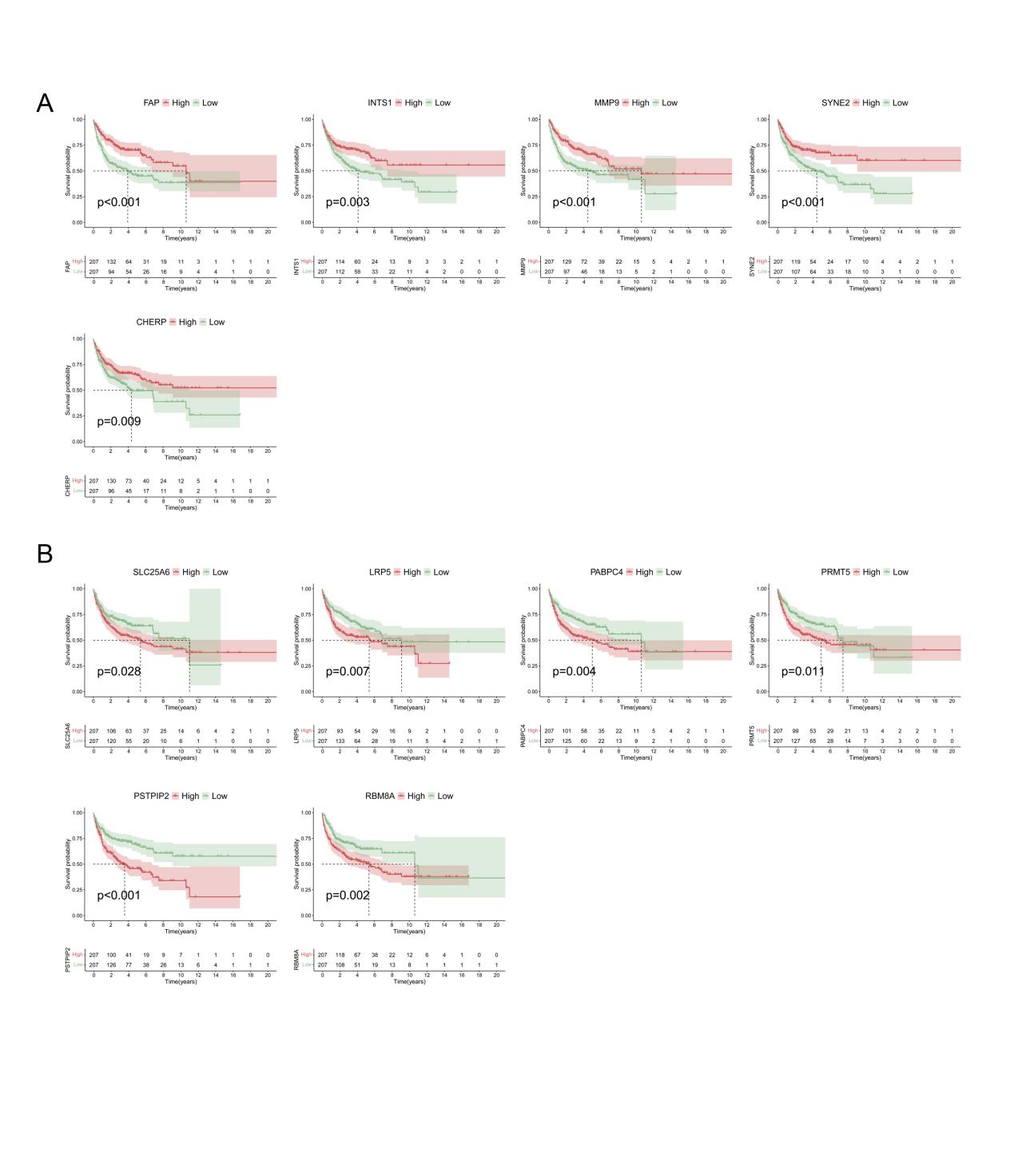


**Supplementary Fig 4.**

**
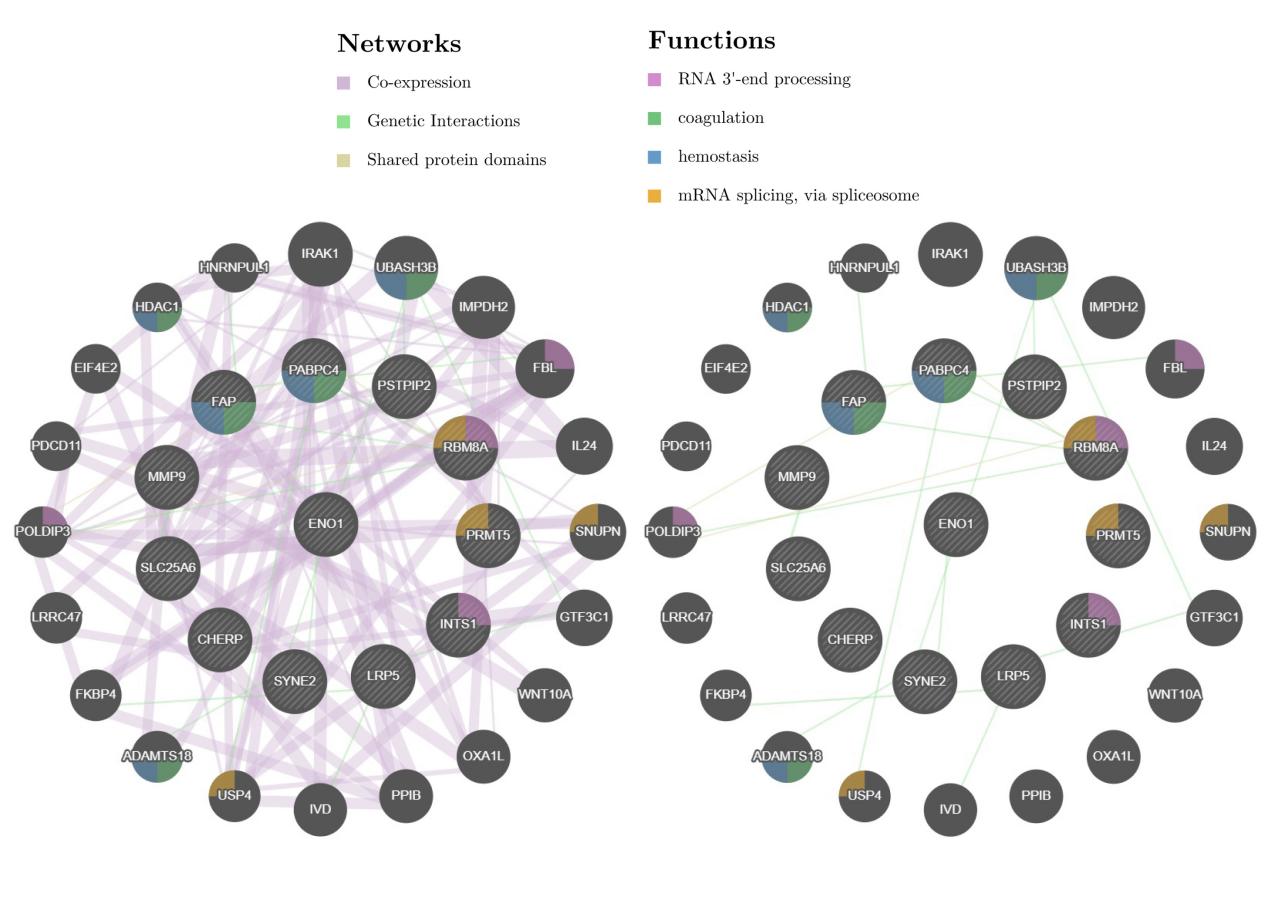
**

**Supplementary Fig 5.**


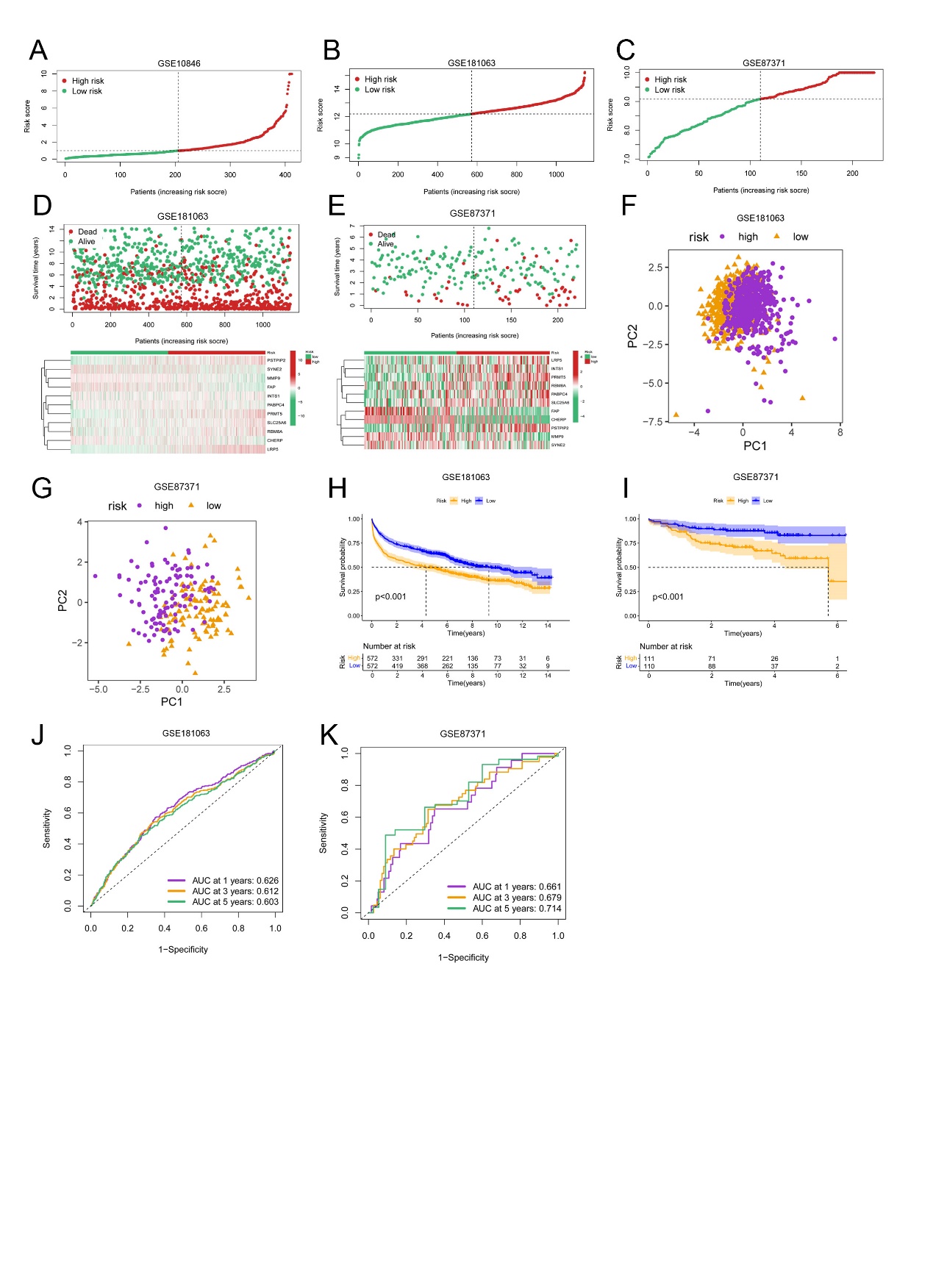


**Supplementary Fig 6.**


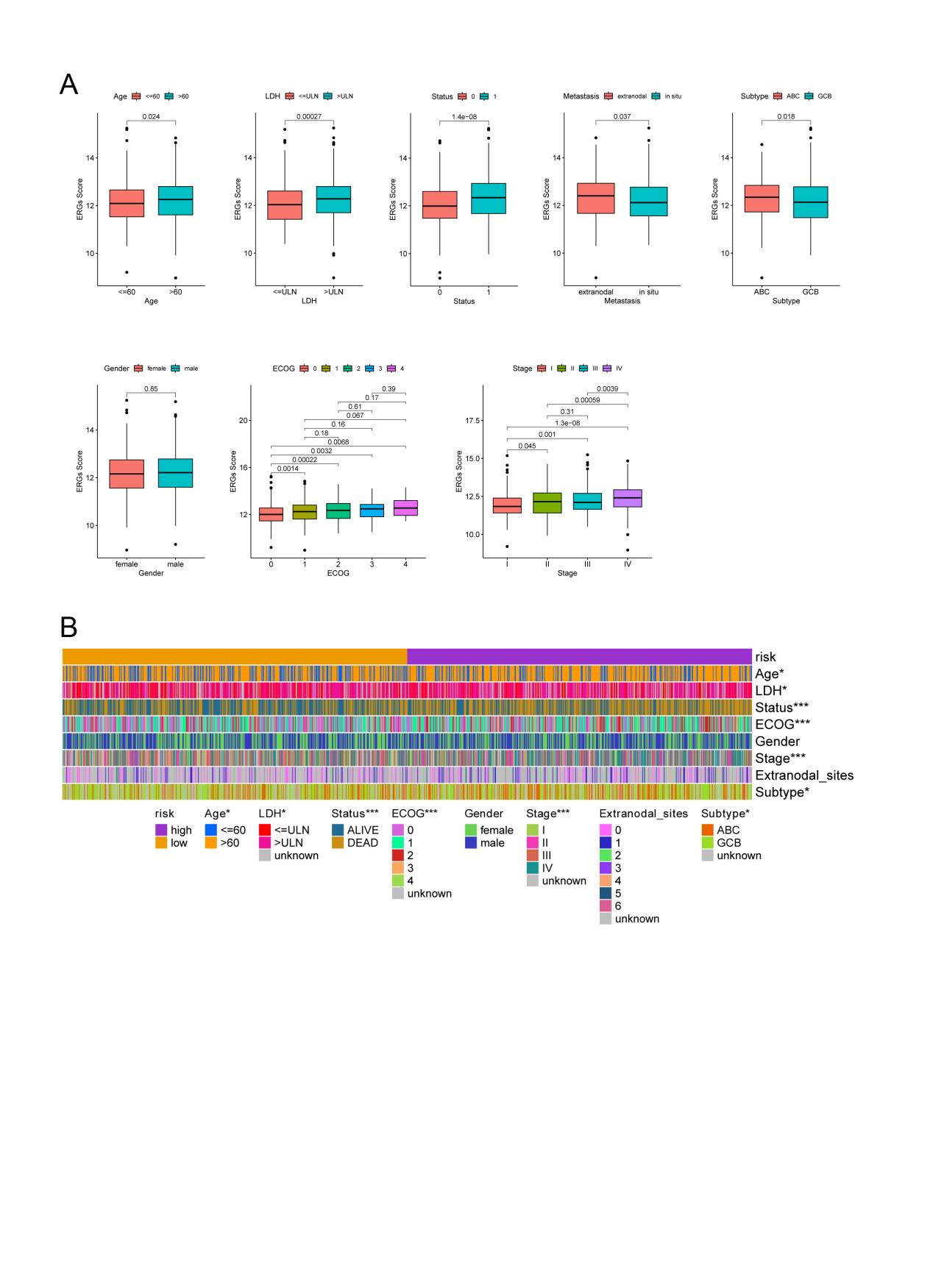


**Supplementary Fig 7.**


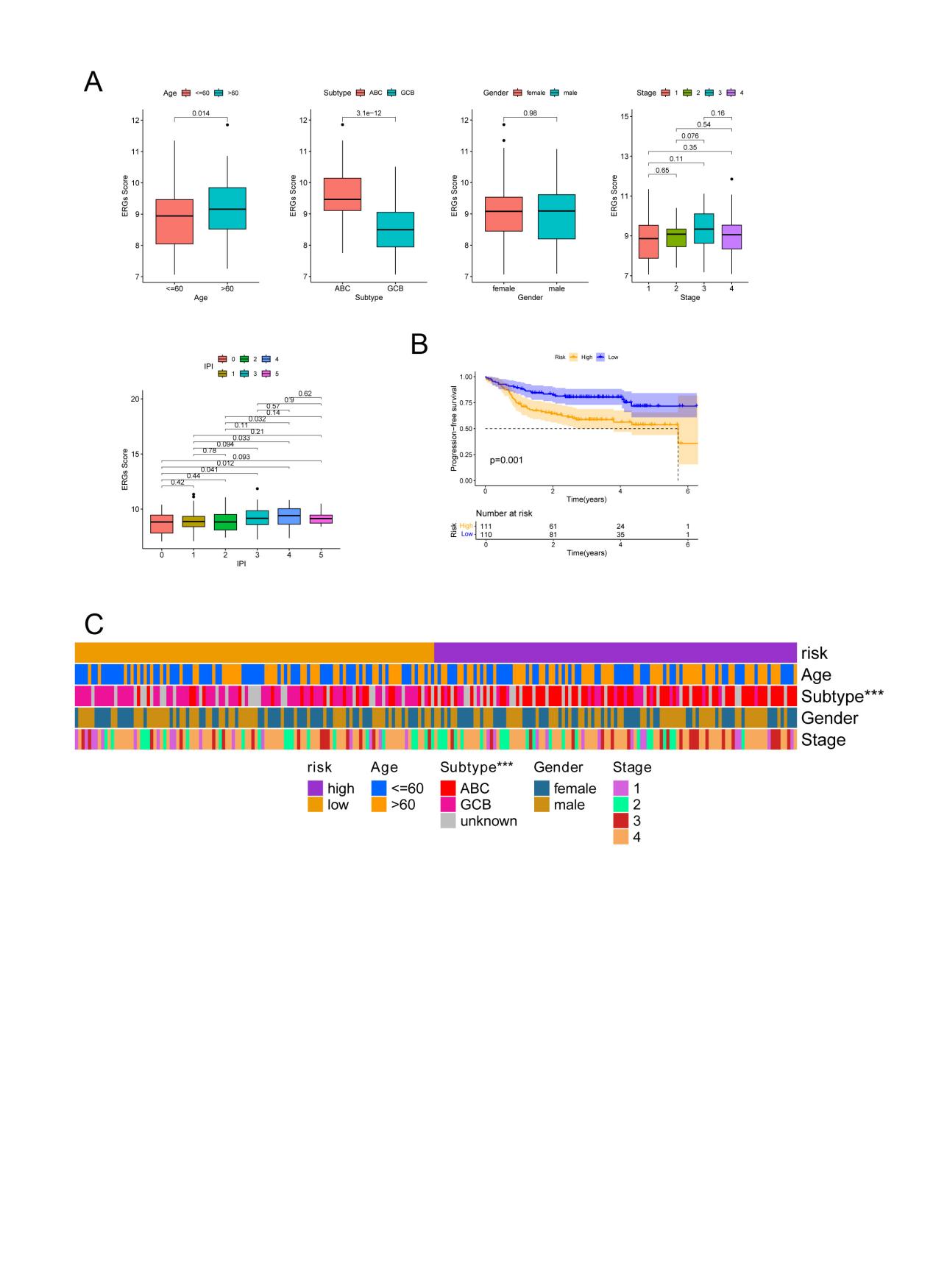


**Supplementary Fig 8.**


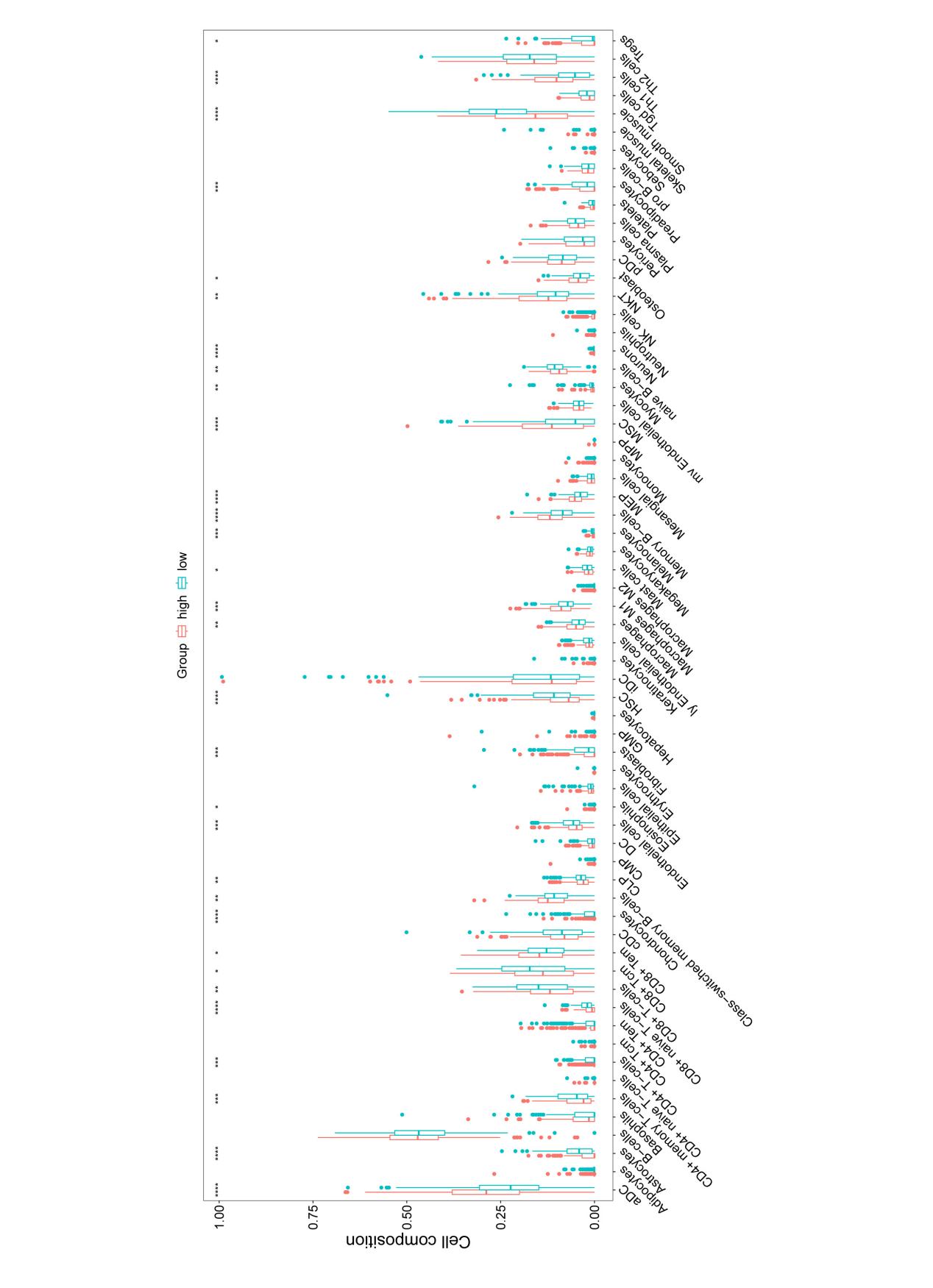


**Supplementary Fig 9.**

**
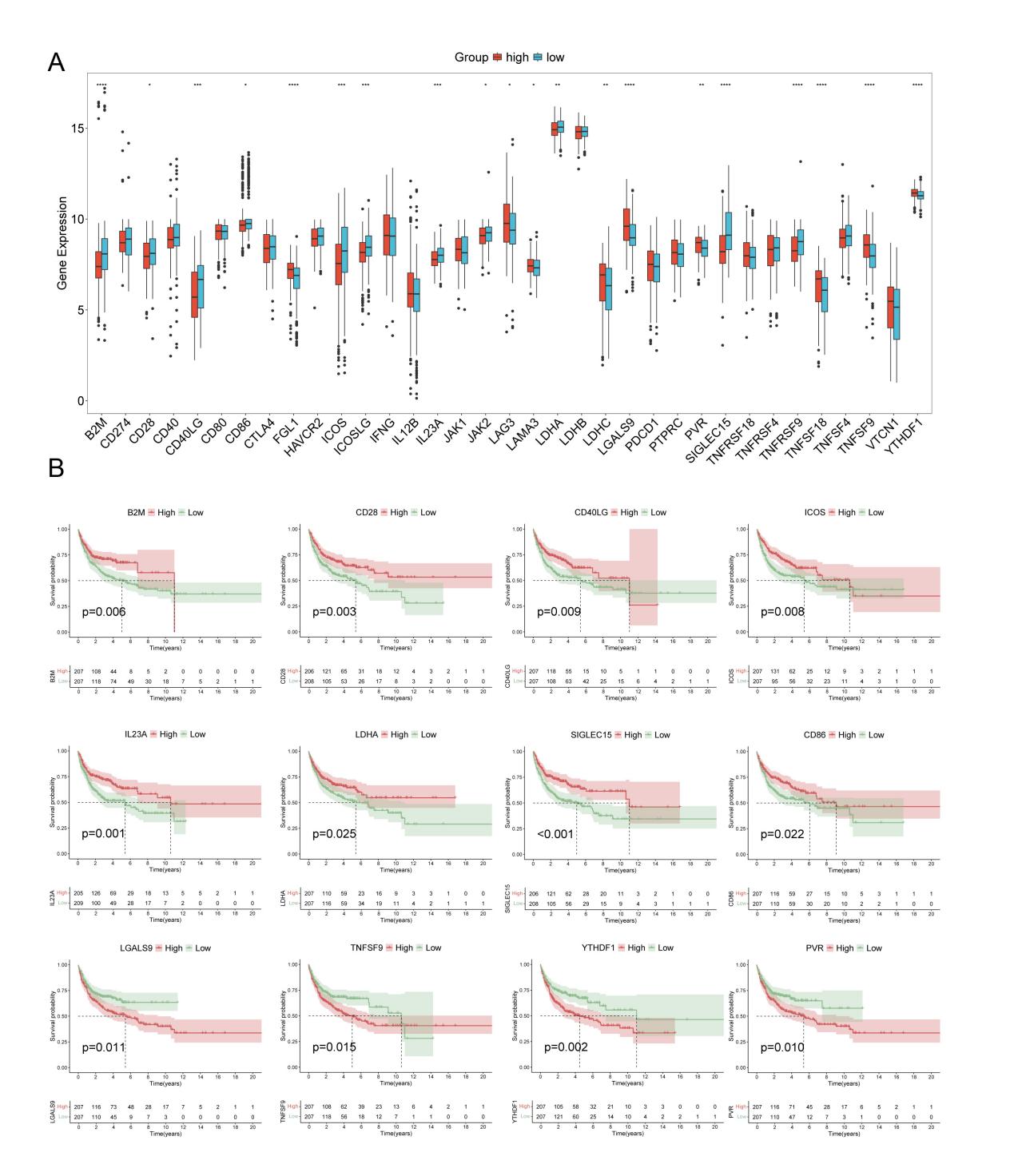
**

**Supplementary Fig 10.**

**
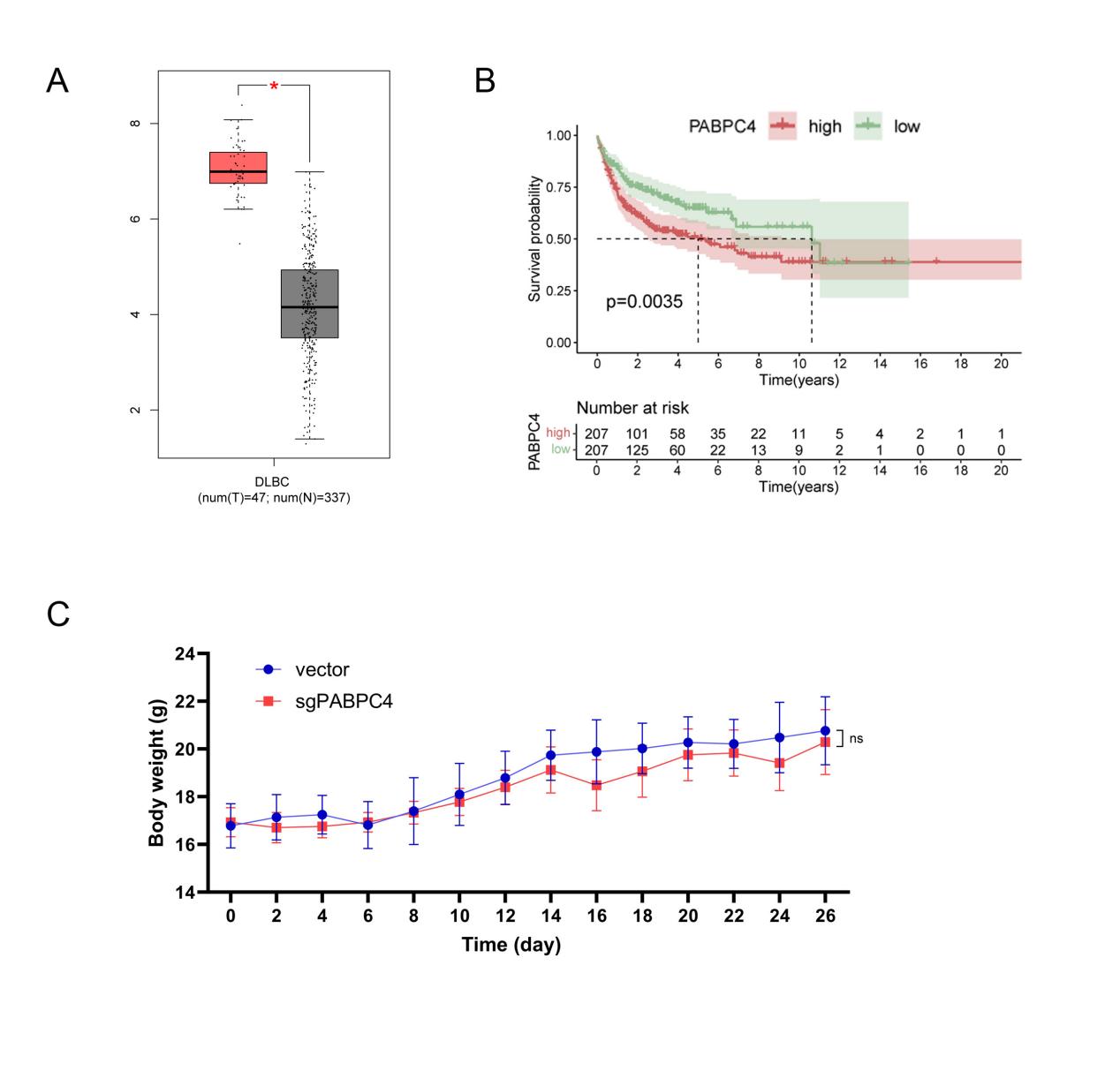
**

**Supplementary Table Legends**

**Supplementary Table 1.** Source and p-values of the 11 ERG genes.

**Supplementary Table 2.** GO and KEGG analysis of the ENO1 and 11 ERG genes.

**Supplementary Table 3.** Univariate and multivariate Cox regression analysis of the ERGs Score and clinical features in GSE10846 dataset.

**Supplementary Table 4.** The expression levels of the 11 prognostic genes in 6 DLBCL cell lines. Cell lines were divided into high- and low-risk group based on the corresponding coefficient of each gene.

**Supplementary Tables**

**Supplementary Table 1.**


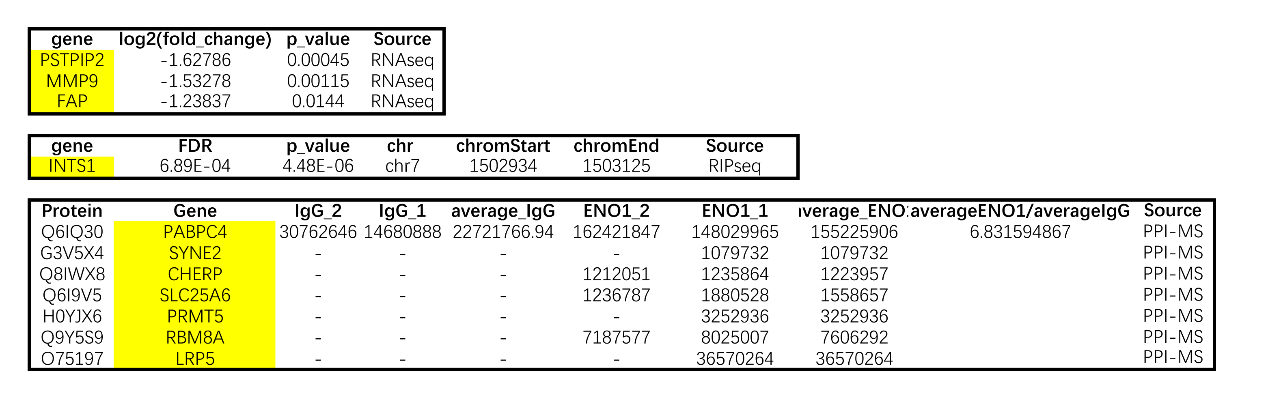


**Supplementary Table 2.**


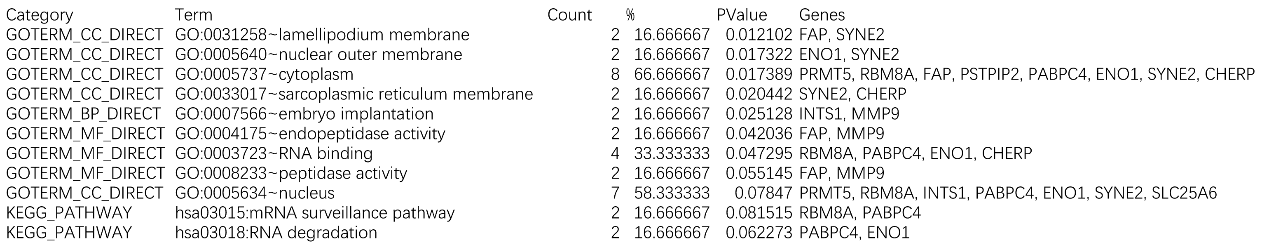


**Supplementary Table 3.**


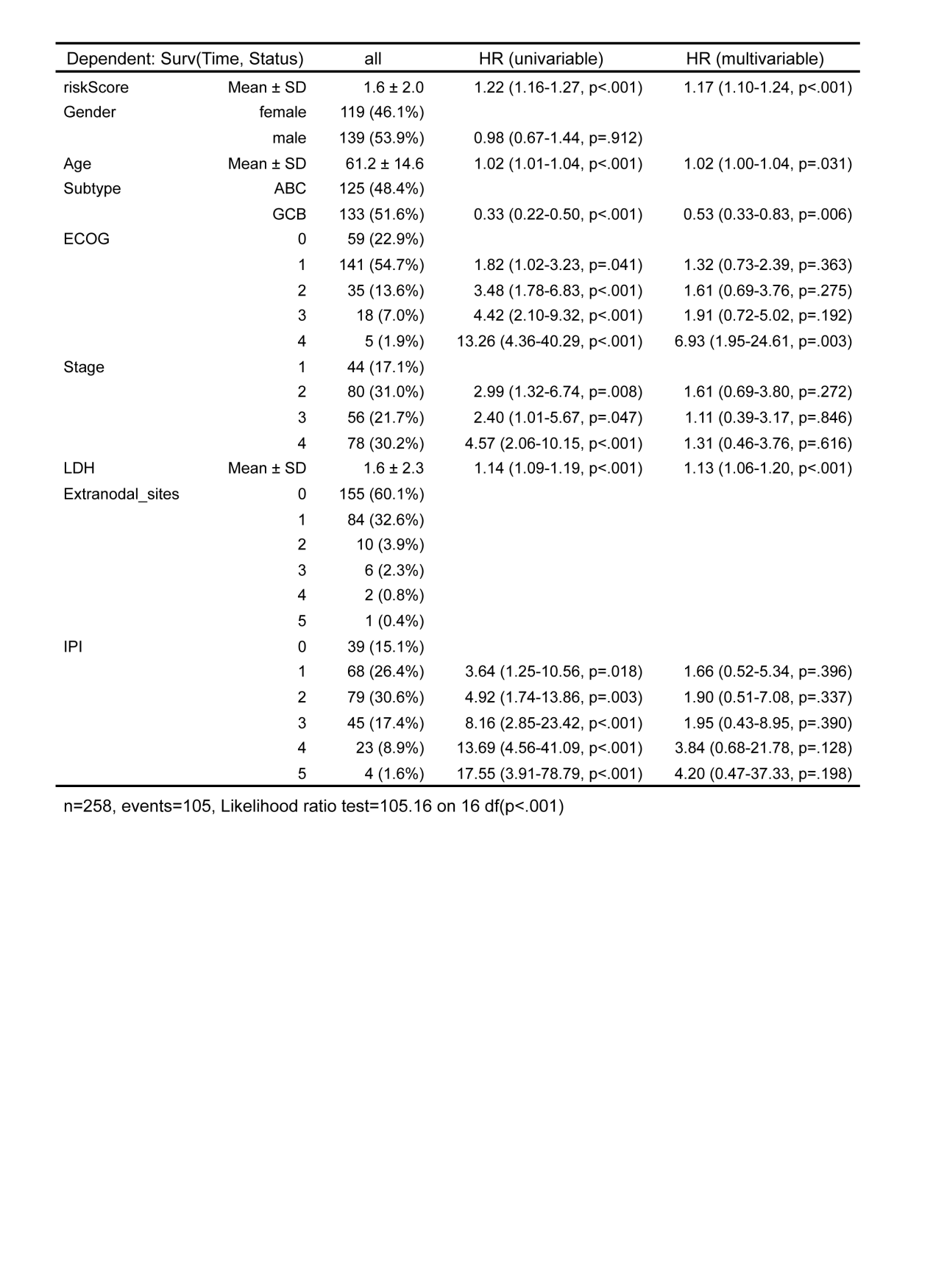


**Supplementary Table 4.**


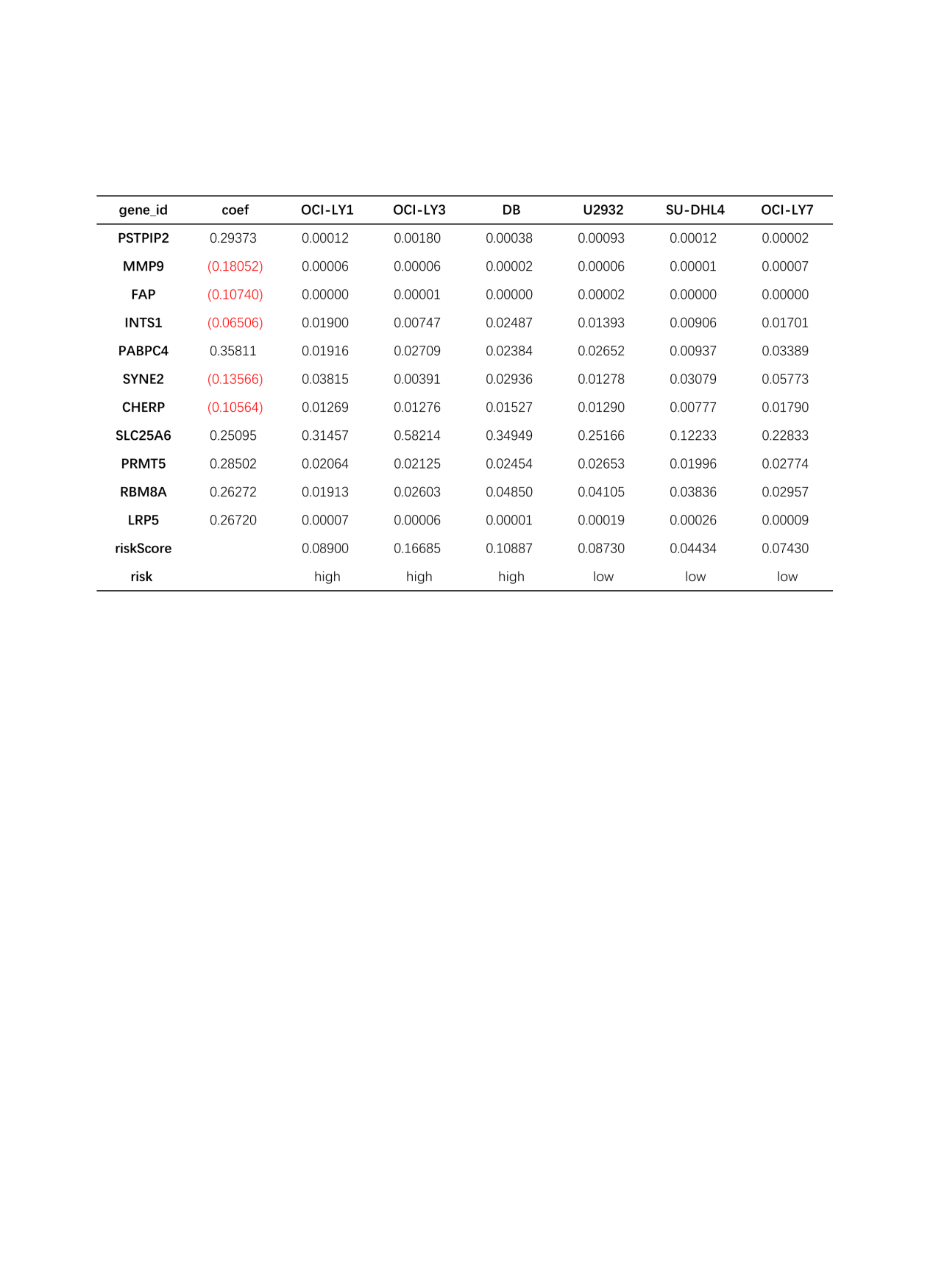

Supplement: Supplementary file 1 [file Table1.docx]
